# Guppy Y Chromosome Integrity Maintained by Incomplete Recombination Suppression

**DOI:** 10.1093/gbe/evaa099

**Published:** 2020-05-19

**Authors:** Iulia Darolti, Alison E Wright, Judith E Mank

**Affiliations:** e1 Biodiversity Research Centre and Department of Zoology, University of British Columbia, Vancouver, British Columbia, Canada; e2 Department of Animal and Plant Sciences, University of Sheffield, Sheffield, United Kingdom; e3 Department of Genetics, Evolution and Environment, University College London, London, United Kingdom

**Keywords:** gametologs, nonrecombining region, sex-linked genes, poeciliid

## Abstract

The loss of recombination triggers divergence between the sex chromosomes and promotes degeneration of the sex-limited chromosome. Several livebearers within the genus *Poecilia* share a male-heterogametic sex chromosome system that is roughly 20 Myr old, with extreme variation in the degree of Y chromosome divergence. In *Poecilia picta*, the Y is highly degenerate and associated with complete X chromosome dosage compensation. In contrast, although recombination is restricted across almost the entire length of the sex chromosomes in *Poecilia reticulata* and *Poecilia wingei*, divergence between the X chromosome and the Y chromosome is very low. This clade therefore offers a unique opportunity to study the forces that accelerate or hinder sex chromosome divergence. We used RNA-seq data from multiple families of both *P. reticulata* and *P. wingei*, the species with low levels of sex chromosome divergence, to differentiate X and Y coding sequences based on sex-limited SNP inheritance. Phylogenetic tree analyses reveal that occasional recombination has persisted between the sex chromosomes for much of their length, as X- and Y-linked sequences cluster by species instead of by gametolog. This incomplete recombination suppression maintains the extensive homomorphy observed in these systems. In addition, we see differences between the previously identified strata in the phylogenetic clustering of X–Y orthologs, with those that cluster by chromosome located in the older stratum, the region previously associated with the sex-determining locus. However, recombination arrest appears to have expanded throughout the sex chromosomes more gradually instead of through a stepwise process associated with inversions.

## Introduction

A common feature of sex chromosomes observed across a diverse array of taxa is loss of recombination, which can ultimately culminate in extreme differences in size and gene content between the sex chromosomes (Bull 1983; Charlesworth et al. 2005; Bachtrog et al. 2011). Nonrecombining regions experience a reduction in the efficiency of selection to remove deleterious mutations as a result of decreased effective population size and accentuated Hill–Robertson effects (Charlesworth and Charlesworth 2000; Charlesworth et al. 2005). As a consequence, over time, initially identical sex chromosomes are expected to diverge from each other in gene content and nucleotide sequence as the sex-limited chromosome accumulates deleterious mutations and degenerates (Charlesworth and Charlesworth 2000; Bachtrog 2013).

It is clear, however, that the degree of sex chromosome divergence does not always correlate with age (Stock et al. 2011; Bachtrog et al. 2014), and therefore Y degeneration is not inevitable, nor is the rate predictable. Because of this, one of the persistent mysteries of sex chromosome evolution is why some X and Y chromosomes show extensive divergence from each other, whereas other systems remain homomorphic (Bachtrog et al. 2014).

Variation in the rate of Y chromosome divergence across taxa could be the effect of different processes leading to recombination suppression. Recombination arrest is thought to initially cover the region containing the sex-determining locus and subsequently expand over larger portions of the sex chromosomes (Charlesworth et al. 2005). The expansion of the nonrecombining region can occur in a stepwise manner, through successive recombination suppression events. This results in distinct regions with different levels of sequence divergence between the gametologs, referred to as evolutionary strata of divergence (Lahn and Page 1999; Charlesworth et al. 2005; Bachtrog 2013). Intrachromosomal rearrangements such as large-scale inversions could result in rapid Y chromosome decay. This mechanism of recombination suppression, often assumed to be the main driver of sex chromosome divergence, would instantaneously prevent recombination throughout the inverted region (Charlesworth et al. 2005). Indeed, sex chromosome divergence in some older systems shows the expected signatures of strata formation via inversions, namely clusters of gametologs with similar divergence estimates (Lahn and Page 1999; Handley et al. 2004; Wright et al. 2012, 2014; Cortez et al. 2014).

However, newly evolved systems show less support for the classic model of sex chromosome evolution (Chibalina and Filatov 2011; Muyle et al. 2012; Roesti et al. 2013). In many younger systems, sequence divergence and recombination suppression between the gametologs occur heterogeneously across the length of the sex chromosomes, suggesting that recombination suppression is a more gradual process than would be expected from the fixation of inversions (Nicolas et al. 2004; Chibalina and Filatov 2011; Bergero et al. 2013; Natri et al. 2013; Almeida et al. 2019). Moreover, if recombination suppression occurs primarily via mechanisms other than inversions, infrequent X–Y recombination events could persist and prevent the sex-limited chromosome from degenerating (Stock et al. 2011, 2013). This permeability of recombination suppression can act to obscure the boundaries between strata, as well as between the nonrecombining region and the PAR (Chibalina and Filatov 2011).

Previous studies have used RNA-seq data and analyses of SNP segregation patterns in families to isolate sex-linked genes from autosomal or pseudoautosomal genes and obtain gametologous (X and Y) sequences (Chibalina and Filatov 2011; Hough et al. 2014; Muyle et al. 2017, 2018; Martin et al. 2019; Veltsos et al. 2019). This approach makes it possible to test for the presence of evolutionary strata by estimating divergence between gametologs and identifying clusters of sex-linked genes with different divergence rates (Lahn and Page 1999; Ross et al. 2005; Wright et al. 2014). Although these analyses can be used to date recombination suppression events and distinguish between the different evolutionary strata of heteromorphic sex chromosomes, they are also useful to define the boundaries between the PAR and the nonrecombining regions of younger, less differentiated systems (Campos et al. 2017).

The common guppy (*Poecilia reticulata*) and its sister species, the Endler’s guppy (*Poecilia wingei*), share the same male-heterogametic sex chromosome system (Morris et al. 2018; Darolti et al. 2019). Our previous analysis of coverage and polymorphism data in males and females revealed evidence of two candidate evolutionary strata in both species. Stratum I, likely predating the divergence of *P. reticulata* and *P. wingei* (Wright et al. 2017; Darolti et al. 2019), corresponds to an area around the sex-determining locus where recombination in males has previously been undetectable (Winge 1922, 1927; Yamamoto 1975), and exhibits mild Y degeneration (Wright et al. 2017; Darolti et al. 2019). Stratum II varies both within and between these two species, and exhibits very rare X–Y recombination events (Winge 1922, 1927; Yamamoto 1975) with the vast majority of male recombination confined to the ends of the chromosome (Bergero et al. 2019). Despite occasional recombination, we previously detected low levels of X–Y divergence in Stratum II (Wright et al. 2017; Darolti et al. 2019), suggesting that either recombinants are selected against, or that the rate of recombination is sufficiently low that it does not fully counter the accumulation of Y chromosome mutations.

Furthermore, although recombination suppression was initially assumed to be quite recent based on the observed low level of divergence between the X and Y chromosomes, we recently showed that this sex chromosome system is in fact far older than expected, as it predates the common ancestor with *Poecilia picta*, roughly 20 Ma (Meredith et al. 2011; Darolti et al. 2019). Curiously, despite the low levels of X and Y differentiation in *P. reticulata* and *P. wingei* (Wright et al. 2017; Morris et al. 2018; Darolti et al. 2019), in *P. picta* the Y is highly diverged from the X and demonstrates complete X chromosome dosage compensation in males (Darolti et al. 2019). This clade with a shared sex chromosome system and a range of sex chromosome divergence presents a unique comparative opportunity to understand the forces that accelerate or retard Y divergence.

Here, we use the probability-based approach, SEX-DETector (Muyle et al. 2016), to infer autosomal and sex-linked genes in *P. reticulata* and *P. wingei* based on RNA-seq data from families. We used phylogenetic and sequence divergence analyses of these sex-linked genes to further characterize the previously defined evolutionary strata and to identify the forces that prevent large-scale sex chromosome degeneration over long evolutionary time. We found significantly more sex-linked genes in *P. wingei* than in *P. reticulata*, consistent with the expansion of the nonrecombining region in the former. The previously defined nonrecombining regions are significantly enriched in sex-linked genes, and we found evidence of recombination suppression before the separation of these two species for four genes in Stratum I. However, the X–Y sequence divergence between genes in the two evolutionary strata is not significantly different, and the same is true between genes in the PAR versus the nonrecombining region, suggesting that recombination arrest has evolved gradually. A phylogenetic analysis of X- and Y-linked sequences reveals that the extensive homomorphism of the poeciliid sex chromosomes is maintained by incomplete recombination suppression. Taken together, our results show that although recombination is largely suppressed across the entire length of the X and Y chromosomes, rare recombination events maintain the integrity of Y coding sequence and expression by preventing large-scale degradation of Stratum II. Our results present an integrated view of how occasional recombination events can retard divergence of sex chromosomes and maintain homomorphy.

## Materials and Methods

### Sample Collection and Sequencing

We established three *P. reticulata* and two *P. wingei* families, and sampled parents and F1 progeny. For generating each family, we used a male and a virgin female either from a *P. reticulata* outbred laboratory population descended from a high predation population of the Quare River, Trinidad (Kotrschal et al. 2013), or from our *P. wingei* laboratory population, derived from a strain maintained by a UK fish hobbyist. We only sampled families where the number of F1 progeny included at least five male and five female individuals, as this is the minimum number of offspring per family required to reliably identify sex-linked genes using the SEX-DETector software (Muyle et al. 2016).

From each individual, we collected the posterior region of the fish behind the anal fin, which we preserved in RNAlater at −20 °C prior to RNA preparation. We extracted RNA from these samples using the RNeasy Kit (Qiagen), following the manufacturer’s instructions with an on-column DNase treatment. Libraries were prepared at the SciLife Lab in Uppsala, Sweden, following standard protocol. RNA was sequenced on an Illumina HiSeq 2500 platform with 125-bp paired-end reads, resulting in an average of 43 million (*P. reticulata*) and 41 million (*P. wingei*) RNA-seq reads per sample. We assessed sample quality using FastQC v0.11.4 (http://www.bioinformatics.babraham.ac.uk/projects/fastqc/, last accessed May 25, 2020) followed by adaptor removal and trimming using Trimmomatic v0.36 (Lohse et al. 2012). We trimmed regions with average Phred score <15 in sliding windows of four bases, reads with Phred score <3 for leading and trailing bases, as well as paired-end reads with either read pair shorter than 50 bp. Following trimming, we had, on an average per sample, 29 million reads for *P. reticulata* and 28 million reads for *P. wingei* (supplementary table 1, Supplementary Material online).

### Constructing and Filtering De Novo Transcriptome Assemblies

We pooled reads from all samples of each species (*n* = 36 for *P. reticulata*; *n* = 24 for *P. wingei*) to construct species-specific de novo transcriptome assemblies (supplementary table 2, Supplementary Material online) using Trinity v2.5.1 (Grabherr et al. 2011) with default parameters, as per previously implemented methods (Harrison et al. 2015; Bloch et al. 2018; Wright, Rogers et al. 2019). We then filtered the assemblies to remove redundancy and noncoding RNA. First, we used the Trinity align_and_estimate_abundance.pl script which maps RNA-seq reads to the transcriptomes using Bowtie2 (Langmead et al. 2009), suppressing unpaired and discordant alignments, and estimates transcript abundance for each sample using RSEM v1.2.25 (Li and Dewey 2011). We then selected the best isoform for each gene based on the highest average expression. In cases where multiple isoforms had the highest expression, we chose the longest isoform as the best isoform. We further filtered the assemblies for noncoding RNA by removing transcripts with a BLAST hit to *Poecilia formosa* (PoeFor_5.1.2) or *Oryzias latipes* (MEDAKA1) ncRNA sequences obtained from Ensembl 84 (Flicek et al. 2014). Lastly, we used Transdecoder v5.2.0 (http://transdecoder.github.io, last accessed May 25, 2020) with default parameters to remove transcripts missing an open-reading frame and transcripts with open-reading frames shorter than 150 bp.

### Assigning Chromosomal Position

We downloaded *P. reticulata* genes from NCBI RefSeq (Guppy_female_1.0+MT, RefSeq assembly accession: GCF_000633615.1) and identified the longest isoform for each gene. We BLASTed the best isoform sequences against the filtered *P. reticulata* and *P. wingei* transcriptome assemblies using BlastN v2.3.0 (Altschul et al. 1990) with an e-value cutoff of 10e^−10^ and a 30% minimum percentage identity. For genes mapping to multiple de novo transcripts, we selected the top BLAST hit based on the highest BLAST bit-score, a measure of sequence similarity. We assigned positional information on *P. reticulata* chromosomes to *P. reticulata* and *P. wingei* transcripts, based on the chromosomal location of genes in the reference.

Previously (Darolti et al. 2019), we have generated pairwise alignments between the *P. reticulata* reference genome and female de novo genome assemblies of *P. wingei* and *P. picta* using LASTZ v1.04 (Harris 2007) and the UCSC chains and nets pipeline (Kent et al. 2003). We recovered a region of the sex chromosome that is inverted in *P. reticulata* relative to *P. wingei* and *P. picta* (supplementary fig. 1, Supplementary Material online). As this analysis was run on female data alone, we inferred this inverted region to be on the X chromosome rather than the Y. It is likely that this inversion has occurred once in *P. reticulata* instead of independently in *P. wingei* and *P. picta*, and is specific to the inbred strain on which the reference genome assembly was built. Considering this, here, we ensured to account for the coordinates of the discovered inversion when assigning de novo transcripts with positional information on the *P. reticulata* sex chromosomes.

### Inferring Autosomal and Sex-Linked Genes

In order to identify sex-linked genes, we used the probability-based method SEX-DETector (Muyle et al. 2016), which analyses the genotypes of individuals in a cross (parents and male and female offspring) to infer the segregation mode of each contig. The model can distinguish between three segregation types: autosomal, sex-linked with both the X and Y copies present (X/Y pair), and sex-linked with the X copy present but the Y copy lost or lowly expressed (X-hemizygous mode). For identifying sex-linked genes with X and Y alleles, SEX-DETector requires gametologs to coassemble in one single transcript instead of separate transcripts. Coassembly makes it possible to identify X/Y SNPs and is necessary to differentiate Y-linked sequences from autosomal genes with male-limited expression. Thus, the SEX-DETector algorithm can more effectively detect X/Y pairs in sex chromosome systems that have low or intermediate levels of divergence. Although X-hemizygous contigs in old systems can be identified using this method, some may in fact be X/Y pairs whose sequences were assembled into separate contigs due to high levels of divergence (Muyle et al. 2016). To avoid assembly of X and Y copies of the same transcript into separate contigs and prevent wrongly inferring contigs as X-hemizygous, we used CAP3 (Huang and Madan 1999), with a 90% minimum percent similarity between sequences, to further assemble contigs. The final filtered transcriptome assemblies contained a total of 19,935 *P. reticulata* transcripts and 19,361 *P. wingei* transcripts (supplementary table 2, Supplementary Material online). The resulting *P. reticulata* and *P. wingei* assemblies are of equivalent quality (supplementary table 2, Supplementary Material online), which gives us a similar power to detect sex-linked loci in these two species.

Based on the SEX-DETector pipeline, we mapped reads onto the filtered assemblies using BWA v0.7.12 (Li and Durbin 2009) and then merged and sorted all libraries of each family separately using SAMtools v1.3.1 (Li et al. 2009). We genotyped individuals at each locus using reads2snp v2.0 (http://kimura.univ-montp2.fr/PopPhyl/, last accessed May 25, 2020), with a minimum number of three reads for calling a genotype (option -min 3), a minimum base quality of 20 (option -bqt 20), a minimum mapping quality of 10 (option -rqt 10), the -aeb option for allowing alleles to have different expression levels, which is important for sex chromosome analyses as the Y copy can be less expressed than the X copy, and the paraclean option disabled (option -par 0) to avoid removal of paralogous positions since X and Y copies can resemble paralogs. We then used SEX-DETector to infer within each family the segregation type for each transcript, using a minimum posterior segregation type probability of 0.8, and to obtain for each family X and Y sequences of each sex-linked gene.

All subsequent analyses were run on a sex-linked gene data set for each species comprised of the pooled sex-linked loci across replicate families. In the case of genes identified as sex-linked in multiple families, we selected the X and Y sequence pairs that contained the highest number of SNP differences as the representative sex-linked sequences for that species. This has increased the power of our downstream analyses, in particular of the divergence estimates for X- and Y-linked gametologs.

### Phylogenetic Analysis

In addition to the *P. reticulata* and *P. wingei* de novo transcript sequences, we obtained *O. latipes* (MEDAKA1), *Xiphophorus maculatus* (Xipmac4.4.2), and *P. formosa* (PoeFor_5.1.2) transcripts from Ensembl 84 and identified the longest transcript for each gene. We determined orthology across all these species using a reciprocal BlastN with an e-value cutoff of 10e^−10^ and a minimum percentage identity of 30%. We then used BlastX to identify open-reading frames in each orthologous group.

We conducted a phylogenetic analysis of X- and Y-linked sequences together with their orthologs in outgroup species to investigate the history of recombination suppression on the sex chromosomes. First, we aligned sequences using PRANK v140603 (Löytynoja and Goldman 2008) and removed gaps in the alignments. We generated maximum likelihood phylogenetic trees with RAxML v8.2.12 (Stamatakis 2014), using the rapid bootstrap algorithm with the GTRGAMMA model and 100 bootstraps. We used Geneious v2019.2.3 (Kearse et al. 2012) to concatenate the alignment files of sex-linked loci in both *P. reticulata* and *P. wingei* into a single file. Using this concatenated file, we ran RAxML using the rapid bootstrap algorithm with the GTRGAMMA model and 100 bootstraps to obtain consensus phylogenetic trees. Additionally, we repeated the phylogenetic analysis by constructing maximum likelihood phylogenetic trees using the GTRGAMMA model and 100 bootstrap replications in MEGA X (Kumar et al. 2018). We used FigTree v1.4.4 (http://tree.bio.ed.ac.uk/software/figtree/, last accessed May 25, 2020) to plot all phylogenetic trees.

### Rates of Evolution of Sex-Linked Genes

For each identified sex-linked gene, we estimated synonymous X–Y divergence (d*S*_XY_) using the *yn00* program in PAML v4.8 (Yang 2007), following the Yang and Nielsen method (Yang and Nielsen 2000). Within each species, we tested for differences in d*S*_XY_ between different gene categories (genes on the PAR 0–15 Mb, the entire nonrecombining region 15–26 Mb, Stratum I 21–26 Mb, or Stratum II 15–21 Mb) and between the species using Wilcoxon rank sum tests in R (R Core Team 2015).

We also estimated rates of evolution of X and Y sequences based on the multiple sequence alignments described earlier. We first masked poorly aligned regions with SWAMP v31-03-14 (Harrison et al. 2014). We then used branch model 2 from the CODEML package in PAML to estimate rates of nonsynonymous (d*N*) and synonymous (d*S*) substitutions for the X- and Y-linked branches in each tree. We used the inferred phylogenetic trees described earlier to generate an unrooted tree for each orthologous group, which is required in the CODEML analyses. We used 1,000 permutation test replicates to identify significant differences in d*N*/d*S* ratios between the X and Y sequences, between the gene categories and between the two species.

### Expression of X- and Y-Linked Genes

Within each species, we mapped reads from each male individual to the identified X- and Y-linked gene sequences and estimated transcript abundance for homologous X- and Y-linked genes using RSEM v1.2.25 (Li and Dewey 2011). For each individual, we calculated Y/X expression at each sex-linked gene and then obtained average Y/X expression ratio across all male individuals of that species.

## Results

We used the genotypes of parents and offspring from multiple *P. reticulata* and *P. wingei* families to infer sex-linkage, which includes genes expressed from both the X and Y chromosomes (X/Y genes) and genes only expressed from the X (X0 genes), following the SEX-DETector pipeline (tables 1 and 2). By mapping inferred sex-linked genes to known *P. reticulata* transcripts and assigning them with chromosomal position, we were able to verify that the vast majority of them (83% in *P. reticulata* and 92% in *P. wingei*) are indeed found on the sex chromosome, *P. reticulata* chromosome 12 (tables 1 and 2), indicating our false-positive rate is quite low. Genes inferred to have a sex-linked inheritance pattern that mapped outside the sex chromosomes are distributed across multiple autosomes and a few unplaced scaffolds (supplementary table 3, Supplementary Material online). We found no enrichment of sex-linked genes on any of the autosomes and there is also little overlap between the two species in the autosomes that contain genes with a sex-linked inheritance pattern (supplementary table 3, Supplementary Material online).

**Table 1:** Number of Inferred *Poecilia reticulata* Sex-Linked Genes

Sex-Linked Genes	All Families	Family 1	Family 2	Family 3
Total number	111	60	41	28
On the sex chromosomes	92 (83%)	54 (90%)	33 (81%)	21 (75%)
On the autosomes	13 (12%)	5 (8%)	3 (7%)	5 (18%)
On unplaced scaffolds	6 (5%)	1 (2%)	5 (12%)	2 (7%)

Note.—These numbers consist of both X/Y and X0 genes.

**Table 2:** Number of Inferred *Poecilia wingei* Sex-Linked Genes

Sex-Linked Genes	All Families	Family 1	Family 2
Total number	272	236	179
On the sex chromosomes	249 (92%)	219 (93%)	163 (91%)
On the autosomes	12 (4%)	7 (3%)	7 (4%)
On unplaced scaffolds	11 (4%)	10 (4%)	9 (5%)

Note.—These numbers consist of X/Y gene pairs only as there were no identified X0 genes in *P. wingei*.

For all subsequent analyses, we only used sex-linked genes which map to chromosome 12. Only three genes were inferred to be sex-linked in all three *P. reticulata* families, whereas 142 genes show signatures of sex-linkage in both *P. wingei* families. Given our overall low false-positive rate, and the fact that the small clutch size of these species limits our power to detect sex-linkage in any one family, we pooled all loci identified as sex-linked across replicate families within each species for following analyses.

SEX-DETector inferred only two X0 genes in *P. reticulata* and none in *P. wingei*. Both *P. reticulata* X0 genes are located in the sex chromosome nonrecombining region (one at 24.9 Mb in the previously identified Stratum I and the other at 17.2 Mb in Stratum II). Genetic diversity can affect our likelihood of identifying X-hemizygous genes, as these are identified by the presence of polymorphisms on the X copy. Thus, there is a lower probability of detecting X-hemizygous genes in *P. wingei* compared with *P. reticulata* as the sampled *P. wingei* population is more inbred and has a lower genetic diversity than the sampled *P. reticulata* population.

Although *P. wingei* and *P. reticulata* share the same sex chromosome system (Morris et al. 2018; Darolti et al. 2019), we found more than twice as many sex-linked genes in *P. wingei* compared with *P. reticulata* (tables 1 and 2). Out of the total number of genes on the sex chromosome that were assigned a segregation type by SEX-DETector, only 92 (46.9%) are inferred as sex-linked in *P. reticulata*, compared with 249 (80.3%) in *P. wingei*. We also found 42 genes that are inferred as sex-linked in both species and that have orthologs in the outgroup species used in our phylogenetic analysis (supplementary table 4, Supplementary Material online), representing ∼46% and, respectively, 17% of the total number of sex-linked genes identified in *P. reticulata* and *P. wingei*.

**Fig. 1.**
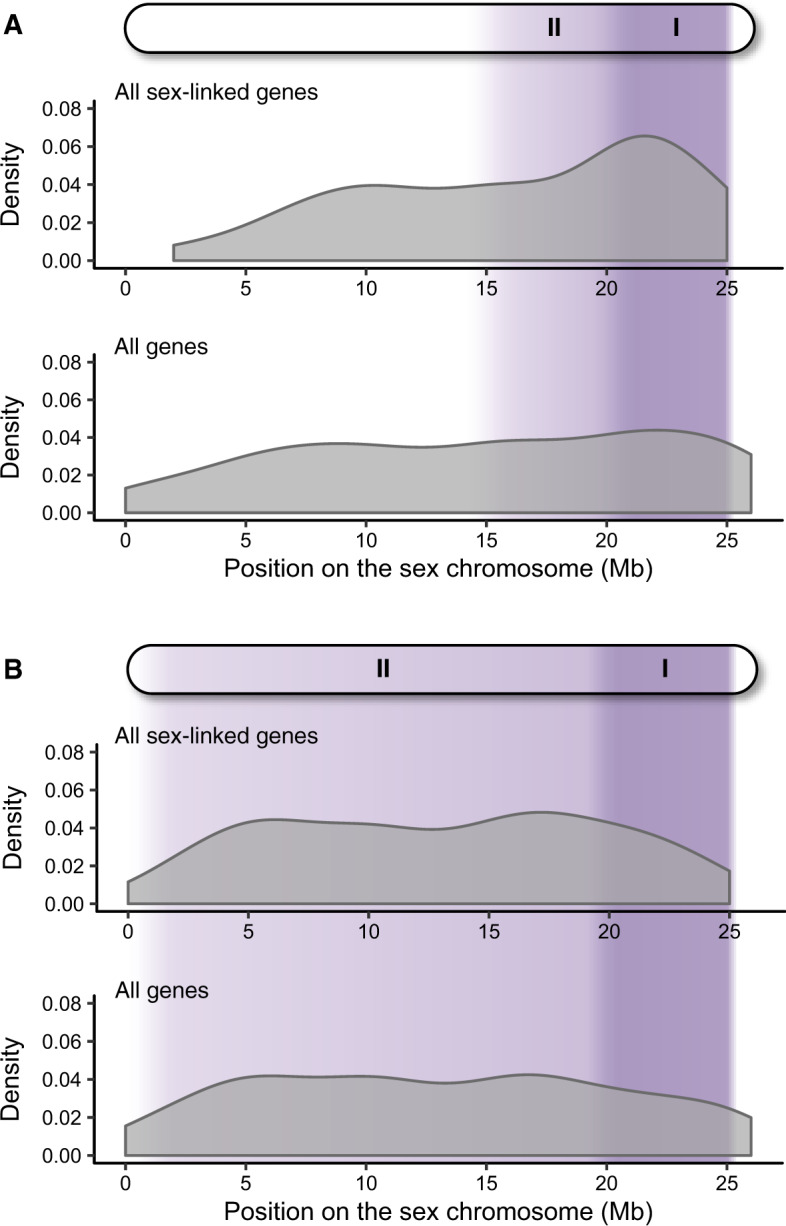
Density of sex-linked genes (top panels) and all genes (bottom panels) across the sex chromosomes of *P. reticulata* (*A*) and *P. wingei* (*B*). The shaded purple regions indicate the previously identified nonrecombining regions (Wright et al. 2017; Darolti et al. 2019). Stratum I is shown in dark purple, where X–Y divergence is the greatest and Stratum II is shown in light purple.

Analyzing the position of all genes along the sex chromosome revealed that sex-linked genes in *P. wingei* are spread throughout the entire length of the sex chromosome, whereas sex-linked genes in *P. reticulata* are predominantly found toward the distal arm of the chromosome (fig. 1 and supplementary figs. 2 and 3, Supplementary Material online). The differences we observe here are consistent with our previous findings based on male:female SNP density, that Stratum II in *P. wingei* appears to extend over a greater proportion of the sex chromosome compared with *P. reticulata* (Darolti et al. 2019). Importantly, we also identified in both species the presence of a limited pseudoautosomal region at the distal end of the chromosome (26–27 Mb), where we recovered many genes with an autosomal inheritance but no sex-linked genes (fig. 1 and supplementary fig. 3, Supplementary Material online), suggesting that recombination persists at a high rate in that area.

Compared with the previously defined pseudoautosomal region, we found a significant enrichment of *P. reticulata* sex-linked genes on the total predicted nonrecombining region of the sex chromosome (Strata I and II together), as well as on each stratum independently (test done using the adjusted strata boundaries described below, Stratum II 15–21 Mb, Stratum I 21–26 Mb; *P *<* *0.02 in all comparisons, Fisher’s exact test; supplementary table 5, Supplementary Material online). The proportion of sex-linked genes, however, does not differ between the two strata in either of the two species (*P *=* *1, odds ratio = 0.94, Fisher’s exact test in *P. reticulata* and *P *=* *1, odds ratio = 1.03, Fisher’s exact test in *P. wingei*). Approximately 36% of the genes on the *P. reticulata* pseudoautosomal region (0–15 Mb) were inferred to be sex-linked.

**Fig. 2.**
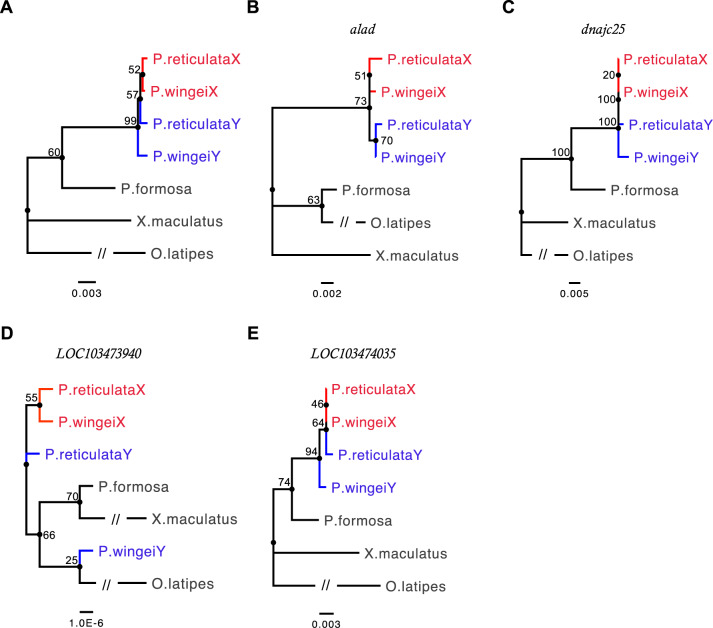
Phylogenetic gene trees for *P. reticulata* and *P. wingei* X- and Y-linked sequences. Phylogenetic trees for the four sex-linked genes in which the X (red) and Y (blue) sequences cluster by gametolog instead of species. (*A*) Consensus tree based on alignments of all four sex-linked genes. Numbers at each node represent bootstrap values based on 100 permutations. Branches with the interrupted lines have been shortened to improve clarity. (*B*) *alad*, (*C*) *dnajc25*, (*D*) *LOC103473940*, and (*E*) *LOC103474035*.

We used the 42 genes identified as sex-linked in both *P. reticulata* and *P. wingei*, together with orthologs in *P. formosa*, *X. maculatus*, and *O. latipes*, in a phylogenetic analysis to investigate recombination suppression on the sex chromosomes. Both phylogenetic analyses reveal clustering of X- and Y-linked sequences by gametolog type instead of by species for four sex-linked genes, suggesting that these genes have stopped recombining before the two species diverged (fig. 2 and supplementary fig. 4, Supplementary Material online). In *P. wingei*, these four sex-linked genes are all found in Stratum I (Darolti et al. 2019), whereas in *P. reticulata*, one of these genes is located in Stratum I, whereas the other three are found at the predicted boundary between the two strata (21–22 Mb) (Wright et al. 2017), suggesting that *P. reticulata* Stratum I is potentially wider than previously estimated based on sequence divergence alone. Considering this, for all our analyses here, we have adjusted the start point of the *P. reticulata* older stratum to 21 Mb. However, in both species, we find that the majority of sex-linked genes cluster by species rather than by chromosome (supplementary figs. 5 and 6, Supplementary Material online).

**Fig. 3.**
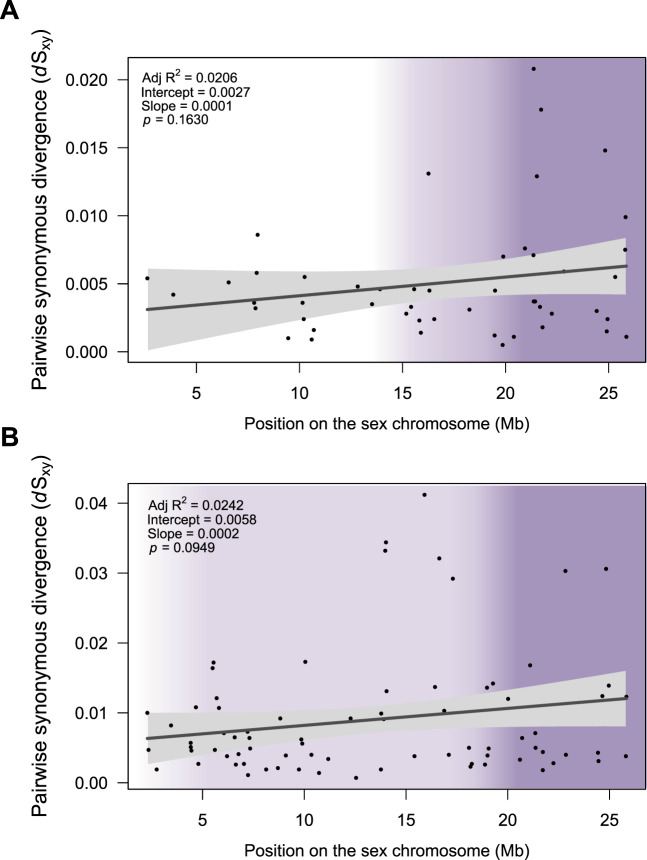
Pairwise synonymous divergence (d*S*_XY_) of *P. reticulata* (*A*) and *P. wingei* (*B*) sex-linked genes across the sex chromosomes. The shaded purple regions indicate the nonrecombining regions. Stratum I is shown in dark purple, where X–Y divergence is the greatest and Stratum II is shown in light purple. Lines show linear regressions fitted to the data using the *lm* function in R and gray-shaded areas represent confidence intervals for the slope of the regression lines.

We estimated the rate of synonymous substitutions (d*S*_XY_) between each pair of X- and Y-linked sequences. Mean d*S*_XY_ is >0 (mean d*S*_XY_ = 0.0059 in *P. reticulata*; mean d*S*_XY_ = 0.1433 in *P. wingei*; supplementary table 6 and fig. 7, Supplementary Material online), and 95% CIs do not overlap with zero. However, we did not observe significant differences in d*S*_XY_ between the PAR, total nonrecombining region, Stratum II, and Stratum I in either species (fig. 3). We found no significant correlation between pairwise synonymous divergence of sex-linked genes and their position on the sex chromosome in either *P. reticulata* (*r*_s_ = 0.14, *P *=* *0.279, Spearman’s rank correlation) or *P. wingei* (*r*_s_ = 0.2, *P *=* *0.095, Spearman’s rank correlation). On average, genes identified as sex-linked in both species have a higher d*S*_XY_ in *P. wingei* than in *P. reticulata*, however, this difference is only marginally significant (median d*S*_XY_ = 0.0045 in *P. reticulata*; median d*S*_XY_ = 0.0057 in *P. wingei*; *P *=* *0.05, Wilcoxon signed rank test).

We also tested whether all identified Y-linked sequences have accumulated more deleterious mutations than X-linked sequences by estimating rates of nonsynonymous (d*N*) and synonymous (d*S*) substitutions and calculating average d*N*/d*S* for each gametolog branch. Overall, Y-linked sequences in both species show a higher d*N*/d*S* compared with X-linked sequences; however, this is not significant (supplementary table 6, Supplementary Material online). These results are in line with our previous findings that neither of these species show Y degeneration or sex differences in transcription of genes on the sex chromosomes (Darolti et al. 2019).

**Fig. 4.**
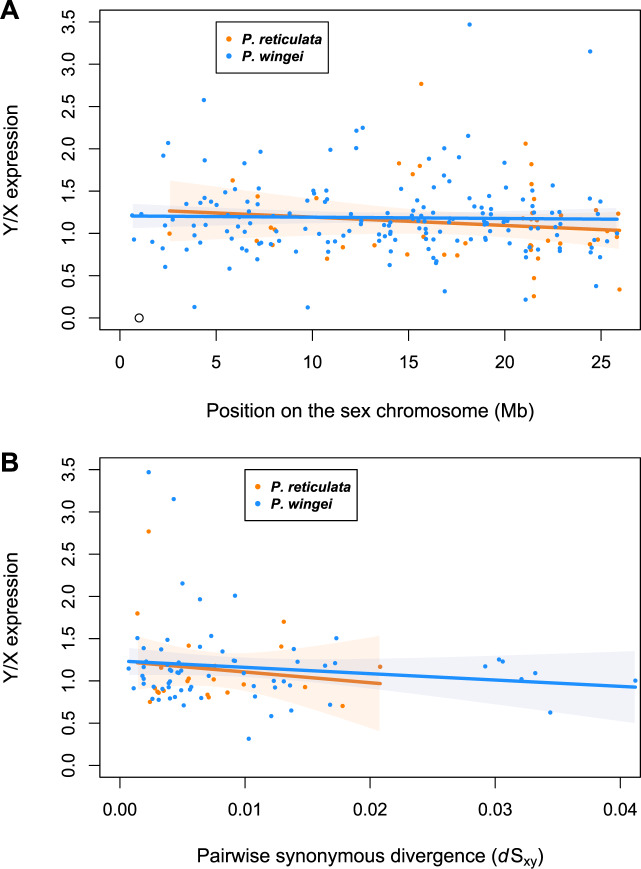
Average male Y/X expression ratio plotted against sex-linked gene position on the sex chromosome (*A*) and pairwise synonymous divergence (d*S*_XY_) (*B*). *Poecilia reticulata* data are represented in orange, whereas *P. wingei* data are in blue. Lines show linear regressions fitted to the data using the *lm* function in R, and orange- and blue-shaded areas represent confidence intervals for the slope of the regression lines.

Compared with homologous X-linked genes, Y-linked genes are expected to gradually decrease in expression following recombination suppression (Chibalina and Filatov 2011). However, for either of the species, we found that average male Y/X expression ratio did not correlate with position on the sex chromosome (fig. 4; adj. *R*^2^ = −0.005, *P *=* *0.376, linear regression slope = −0.010 for *P. reticulata*, adj. *R*^2^= −0.006, *P *=* *0.774, linear regression slope = −0.002 for *P. wingei*) or with pairwise synonymous divergence (fig. 4; adj. *R*^2^ = −0.028, *P *=* *0.518, linear regression slope = −0.002 for *P. reticulata*, adj. *R*^2^ = 0.004, *P *=* *0.260, linear regression slope = −0.003 for *P. wingei*).

## Discussion

Complete recombination suppression over portions of the sex chromosomes is expected over time to favor the accumulation of deleterious mutations and lead to loss of gene activity and function on the sex-limited chromosome (Bachtrog 2013). In spite of these predictions, *P. reticulata* and *P. wingei* share a sex chromosome system that is largely nonrecombining, yet which shows very low X–Y sequence divergence and no reduction of male gene activity and content (Wright et al. 2017; Darolti et al. 2019). These findings lead to questions about the mechanisms maintaining Y sequence integrity in these species. That these species also share a sex chromosome system with *P. picta*, which shows marked Y chromosome degeneration (Darolti et al. 2019), suggests that the mechanism of recombination suppression is far less effective in *P. reticulata* and *P. wingei.*

### Incomplete Recombination Suppression

In the complete absence of recombination, and with sufficient time for complete lineage sorting, we would expect phylogenetic analyses of sex-linked genes to show clustering of X- and Y-linked sequences by gametolog instead of by species. We have previously observed multiple concordant lines of evidence for two strata on the guppy sex chromosome. First, we see clear differences in male:female coverage for Stratum I and male:female SNP density for Stratum II in *P. reticulata* (Wright et al. 2017). Second, we replicated these analyses in *P. wingei* and showed that these strata also exist in this species (Darolti et al. 2019). Third, we see a conservation of male-specific *k*-mers between these species (Morris et al. 2018), indicative of Y divergence in Stratum I. These strata were however not observed by Bergero et al. (2019), most likely due to differences in mapping stringency (Wright, Darolti, et al. 2019).

Consistent with the evolutionary strata, we have previously identified (Wright et al. 2017; Darolti et al. 2019), here, we observe signatures of regions with different time since recombination suppression. Specifically, we observe phylogenetic clustering by sex chromosome in four sex-linked genes (fig. 2 and supplementary fig. 4, Supplementary Material online), suggesting that recombination suppression was finalized before the split of *P. wingei* and *P. reticulata*. Recombination suppression in the common ancestor is also consistent with the substantial amount of shared male-specific sequence that has been found in these species (Morris et al. 2018). These genes are located either in Stratum I or at its predicted boundary with Stratum II (supplementary table 4, Supplementary Material online), allowing us to define the sex chromosome region spanning 21–26 Mb as being the ancestral, more differentiated, nonrecombining region. This is consistent with previous genetic mapping of the sex-determining locus to this region (Tripathi et al. 2009), and the region where X–Y recombination has been previously undetected (Winge 1922, 1927; Yamamoto 1975).

Outside the older nonrecombining region, in the previously identified Stratum II, we find that Y chromosomes in *P. reticulata* and *P. wingei* have a higher sequence similarity to their homologous X regions than to each other (supplementary figs. 5 and 6, Supplementary Material online). This points to either incomplete lineage sorting or incomplete recombination suppression in this system. Although recombination is largely curtailed in males (Bergero et al. 2019), occasional recombination events prevent the rapid degeneration expected of fully nonrecombining regions (Winge 1922, 1927; Yamamoto 1975). Infrequent X–Y recombination in males or in sex-reversed females has been shown to be sufficient to maintain a high level of sequence similarity between gametologs in *Hyla* frogs for up to 7 Myr (Stock et al. 2011, 2013), and has been documented in *P. reticulata* (Haskins et al. 1961; Lisachov et al. 2015) though it has been difficult to quantify the rate or the proportion of recombination nodules that result in strand invasion. That the guppy sex chromosome system is at least 20 Myr old suggests that recombination suppression can remain permeable on sex chromosomes far longer than previously recognized and can act as a long-term and persistent brake of Y chromosome degeneration.

It is also possible that we have underestimated the number of loci that have stopped recombining before *P. reticulata* and *P. wingei* split, as factors such as gene conversion or gene sequence length may cause a false signal of phylogenetic clustering by species. An alternative mechanism acting to prevent neutral degradation of the sex-limited chromosome is gene conversion between gametologs (Slattery et al. 2000; Rosser et al. 2009; Trombetta et al. 2010; Wright et al. 2014). Interchromosomal gene conversion events take place in meiosis when one allele is converted into its homolog in the process of double-strand DNA break repair (Chen et al. 2007). Testing for the presence of gene conversion relies on identifying stretches of X–Y identical sequence delimited by variable sites (Sawyer 1999; Wright et al. 2014). Unfortunately, here, the high similarity between the identified X- and Y-linked sequences in *P. reticulata* and *P. wingei* prevents such analyses.

We note that the lower phylogenetic bootstrap support values for the four single-gene trees (fig. 2*B*–*E*) may be the result of incomplete lineage sorting, persistent gene conversion, and most importantly, from the short coding sequence of these loci. Bootstrap support for phylogenetic approaches such as this are often low, even for loci in significantly older strata (Wright et al. 2014). Additionally, bootstrap support values are expected to be higher for majority consensus trees, which are constructed on alignments of many sets of genes, than for single-gene trees. We observe that to be the case for both sex-linked genes that show clustering by gametolog (fig. 2) and for sex-linked genes that cluster by species (supplementary figs. 5 and 6, Supplementary Material online).

We previously detected low levels of divergence in Stratum II (Wright et al. 2017; Darolti et al. 2019), and our analysis here also finds d*S*_XY_ >0 in this region (fig. 3). However, we previously detected significant accumulation of Y-specific SNPs (Wright et al. 2017; Darolti et al. 2019), suggesting that either recombinants are selected against, or that the rate of recombination is insufficient to fully counter the accumulation of Y mutations. It is worth mentioning that the permeability of recombination suppression found here can obscure additional differences between older and younger regions of recombination suppression.

It is also possible that the degenerate *P. picta* Y chromosome represents the ancestral state, and the homomorphic system in *P. reticulata* and *P. wingei* represents a reversion to homomorphy, as has been recently documented in geckos (Rovatsos et al. 2019). However, the presence of the complete X chromosome dosage compensation observed in *P. picta* (Darolti et al. 2019) makes this unlikely. It has been argued that complete dosage compensation mechanisms will prevent sex chromosome turnover as the heterogametic sex would have two upregulated ancestral X chromosomes (Van Doorn and Kirkpatrick 2010; Mank et al. 2011). Given the substantial gene content of the X chromosome (Kunstner et al. 2016), this would lead to probable lethality due to overexpression of dosage-sensitive genes in males.

### Gradual Expansion of Recombination Suppression

Our recent analyses of genomic coverage and polymorphism data in males and females indicate a larger nonrecombining region in *P. wingei* compared with *P. reticulata* (Wright et al. 2017; Darolti et al. 2019). Indeed, here, we find that the extent of sex-linkage is greater in *P. wingei* compared with *P. reticulata*. However, we also find sex-linked genes present in the previously defined *P. reticulata* pseudoautosomal region, suggesting that recombination suppression in this species may have expanded beyond the previously identified nonrecombining region, but has not yet resulted in significant sequence divergence of the X and Y chromosomes. Alternatively, this may be due to partial linkage of the PAR when a proportion of male recombination events occur somewhat distant from the PAR-sex chromosome boundary. We are also able to confirm the presence of a second small pseudoautosomal region at the distal end of the sex chromosomes (26–27 Mb), region which has been previously suggested to be recombining (Lisachov et al. 2015; Bergero et al. 2019; Darolti et al. 2019).

The results of our sex-linkage analysis suggest that, outside of Stratum I, either recombination suppression has occurred well after the split of *P. reticulata* and *P. wingei*, or recombination suppression is ancestral but has remained incomplete. Given that occasional recombination is still observed in this region (Winge 1922, 1927; Yamamoto 1975), the latter explanation is more likely, and this suggests that recombination suppression is imperfect, and likely not the result of large-scale inversions in this system. In the absence of an inversion, it is likely that the frequency or chromosomal location of these occasional recombination events vary across different populations, consistent with the divergence differences we previously observed in natural populations of *P. reticulata* (Wright et al. 2017, but see Bergero et al. 2019; Wright, Darolti, et al. 2019). Furthermore, population-level differences in the rate of X–Y recombination could explain observed differences in the Y-linkage of guppy color traits (Lindholm and Breden 2002; Gordon et al. 2012).

Although we observe that d*S*_XY_ >0, this is not due to significant increase in rates on the Y chromosome compared with the X (supplementary table 6, Supplementary Material online), and does not differ across sex chromosome regions (fig. 3). Significant variation in pairwise synonymous substitution rates between strata is expected to be mainly seen in old sex chromosome systems where strata ages are very different. Previous studies on younger systems, such as those of *Mercurialis* (Veltsos et al. 2019) and of *Silene* (Papadopulos et al. 2015), show evidence of distinct evolutionary strata yet without significant differences in rates of gene sequence evolution between them. In completely nonrecombining regions, the Y chromosome is expected to undergo a higher mutation rate than the X chromosome. However, incomplete recombination suppression, as observed here in the guppy system, will homogenize mutation rate between the X and the Y chromosomes such that we do not necessarily expect to see a consistently higher rate of mutation on the Y chromosome for all sex-linked genes. The results suggest a more gradual expansion of recombination suppression instead of a stepwise process resulting from inversions. It is worth noting though that discrete evolutionary strata and clear boundaries between the nonrecombining region and the PAR may be masked by the stochastic differences between genes in X–Y divergence resulting from occasional recombination events (Chibalina and Filatov 2011).

## Concluding Remarks

Taken together, our results present a permeable picture of recombination suppression. The sex chromosomes in *P. reticulata* and *P. wingei* arose in the shared ancestral lineage with *P. picta* (Darolti et al. 2019) roughly 20 Ma (Meredith et al. 2011) and have proceeded at markedly different rates. In *P. picta*, the highly degenerate Y chromosome is paired with complete X chromosome dosage compensation. However, in *P. reticulata* and *P. wingei*, which share substantial male-specific sequence (Morris et al. 2018), there has been no perceptible loss of Y chromosome gene activity (Wright et al. 2017; Darolti et al. 2019). Our results indicate that occasional X–Y recombination acts to maintain Y chromosome integrity far longer than previously recognized (Stock et al. 2011), and the degree to which recombination persists may explain the heterogeneity in the rate of Y chromosome degeneration observed across disparate sex chromosome systems (Bachtrog et al. 2014).

## Supplementary Material

evaa099_Supplementary_Data

## Notes

**Data deposition:** RNA-seq reads from this project have been deposited at the NCBI Sequencing Read Archive under the NCBI BioProject accession PRJNA591249.

## References

[evaa099-B1] Almeida P, et al 2019 Single-molecule genome assembly of the basket willow, *Salix viminalis*, reveals earliest stages of sex chromosome expansion. bioRxiv: 589804. 10.1186/s12915-020-00808-1 PMC7329446 32605573

[evaa099-B2] Altschul SF, Gish W, Miller W, Myers E, Lipman DJ. 1990 Basic local alignment search tool. J Mol Biol. 215(3):403–410. 2231712 10.1016/S0022-2836(05)80360-2

[evaa099-B3] Bachtrog D. 2013 Y-chromosome evolution: emerging insights into processes of Y-chromosome degeneration. Nat Rev Genet. 14(2):113–124. 23329112 10.1038/nrg3366 PMC4120474

[evaa099-B4] Bachtrog D, et al 2011 Are all sex chromosomes created equal? Trends Genet. 27(9):350–357. 21962970 10.1016/j.tig.2011.05.005

[evaa099-B5] Bachtrog D, et al 2014 Sex determination: why so many ways of doing it? PLoS Biol. 12(7):e1001899. 24983465 10.1371/journal.pbio.1001899 PMC4077654

[evaa099-B6] Bergero R, Gardner J, Bader B, Yong L, Charlesworth D. 2019 Exaggerated heterochiasmy in a fish with sex-linked male coloration polymorphisms. Proc Natl Acad Sci U S A. 116(14):6924–6931. 30894479 10.1073/pnas.1818486116 PMC6452659

[evaa099-B7] Bergero R, Qiu S, Forrest A, Borthwick H, Charlesworth D. 2013 Expansion of the pseudo-autosomal region and ongoing recombination suppression in the *Silene latifolia* sex chromosomes. Genetics 194(3):673–686. 23733786 10.1534/genetics.113.150755 PMC3697972

[evaa099-B8] Bloch NI, et al 2018 Early neurogenomic response associated with variation in guppy female mate preference. Nat Ecol Evol. 2(11):1772–1781. 30297748 10.1038/s41559-018-0682-4 PMC6349141

[evaa099-B9] Bull JJ. 1983 Evolution of sex determining mechanisms. Menlo Park (CA): The Benjamin /Cummings Publishing Company, Inc.

[evaa099-B10] Campos J, Qiu S, Guirao-Rico S, Bergero R, Charlesworth D. 2017 Recombination changes at the boundaries of fully and partially sex-linked regions between closely related *Silene* species pairs. Heredity 118(4):395–403. 27827389 10.1038/hdy.2016.113 PMC5345606

[evaa099-B11] Charlesworth B, Charlesworth D. 2000 The degenration of Y chromosomes. Philos Trans R Soc Lond B. 355(1403):1563–1572. 11127901 10.1098/rstb.2000.0717 PMC1692900

[evaa099-B12] Charlesworth D, Charlesworth B, Marais G. 2005 Steps in the evolution of heteromorphic sex chromosomes. Heredity 95(2):118–128. 15931241 10.1038/sj.hdy.6800697

[evaa099-B13] Chen J-M, Cooper DN, Chuzhanova N, Férec C, Patrinos GP. 2007 Gene conversion: mechanisms, evolution and human disease. Nat Rev Genet. 8(10):762–775. 17846636 10.1038/nrg2193

[evaa099-B14] Chibalina MV, Filatov DA. 2011 Plant Y chromosome degeneration is retarded by haploid purifying selection. Curr Biol. 21(17):1475–1479. 21889890 10.1016/j.cub.2011.07.045

[evaa099-B15] Cortez D, et al 2014 Origins and functional evolution of Y chromosomes across mammals. Nature 508(7497):488–493. 24759410 10.1038/nature13151

[evaa099-B16] Darolti I, et al 2019 Extreme heterogeneity in sex chromosome differentiation and dosage compensation in livebearers. Proc Natl Acad Sci U S A. 116(38):19031–19036. 31484763 10.1073/pnas.1905298116 PMC6754558

[evaa099-B17] Flicek P, et al 2014 Ensembl 2014. Nucleic Acids Res. 42(D1):749–755.

[evaa099-B18] Gordon SP, Lopez-Sepulcre A, Reznick DN. 2012 Predation-associated differences in sex linkage of wild guppy coloration. Evolution 66(3):912–918. 22380450 10.1111/j.1558-5646.2011.01495.x

[evaa099-B19] Grabherr MG, et al 2011 Trinity: reconstructing a full-length transcriptome without a genome from RNA-Seq data. Nat Biotechnol. 29(7):644–652. 21572440 10.1038/nbt.1883 PMC3571712

[evaa099-B20] Handley L-JL, Ceplitis H, Ellegren H. 2004 Evolutionary strata on the chicken Z chromosome: implications for sex chromosome evolution. Genetics 167(1):367–376. 15166161 10.1534/genetics.167.1.367 PMC1470863

[evaa099-B21] Harris RS. 2007. Improved pairwise alignment of genomic DNA [PhD thesis]. [University Park (PA)]: Pennsylvania State University.

[evaa099-B22] Harrison PW, et al 2015 Sexual selection drives evolution and rapid turnover of male gene expression. Proc Natl Acad Sci U S A. 112(14):4393–4398. 25831521 10.1073/pnas.1501339112 PMC4394296

[evaa099-B23] Harrison PW, Jordan GE, Montgomery SH. 2014 SWAMP: sliding window alignment masker for PAML. Evol Bioinform. 10(EBO):S18193. 10.4137/EBO.S18193 PMC4251194 25525323

[evaa099-B24] Haskins CP, Haskins EF, McLaughlin J, Hewitt R. 1961 Polymorphism and population structure in *Lebistes reticulatus*, an ecological study. Vertebr Speciation. 320:395.

[evaa099-B25] Hough J, Hollister JD, Wang W, Barrett SC, Wright SI. 2014 Genetic degeneration of old and young Y chromosomes in the flowering plant *Rumex hastatulus*. Proc Natl Acad Sci U S A. 111(21):7713–7718. 24825885 10.1073/pnas.1319227111 PMC4040613

[evaa099-B26] Huang X, Madan A. 1999 CAP3: a DNA sequence assembly program. Genome Res. 9(9):868–877. 10508846 10.1101/gr.9.9.868 PMC310812

[evaa099-B27] Kearse M, et al 2012 Geneious Basic: an integrated and extendable desktop software platform for the organization and analysis of sequence data. Bioinformatics 28(12):1647–1649. 22543367 10.1093/bioinformatics/bts199 PMC3371832

[evaa099-B28] Kent WJ, Baertsch R, Hinrichs A, Miller W, Haussler D. 2003 Evolution’s cauldron: duplication, deletion, and rearrangement in the mouse and human genomes. Proc Natl Acad Sci U S A. 100(20):11484–11489. 14500911 10.1073/pnas.1932072100 PMC208784

[evaa099-B29] Kotrschal A, et al 2013 Artificial selection on relative brain size in the guppy reveals costs and benefits of evolving a larger brain. Curr Biol. 23(2):168–171. 23290552 10.1016/j.cub.2012.11.058 PMC3566478

[evaa099-B30] Kumar S, Stecher G, Li M, Knyaz C, Tamura K. 2018 MEGA X: molecular evolutionary genetics analysis across computing platforms. Mol Biol Evol. 35(6):1547–1549. 29722887 10.1093/molbev/msy096 PMC5967553

[evaa099-B31] Kunstner A, et al 2016 The genome of the Trinidadian guppy, *Poecilia reticulata*, and variation in the Guanapo population. PLoS One 11(12):e0169087. 28033408 10.1371/journal.pone.0169087 PMC5199103

[evaa099-B32] Lahn BT, Page DC. 1999 Four evolutionary strata on the human X chromosome. Science 286(5441):964–967. 10542153 10.1126/science.286.5441.964

[evaa099-B33] Langmead B, Trapnell C, Pop M, Salzberg SL. 2009 Ultrafast and memory-efficient alignment of short DNA sequences to the human genome. Genome Biol. 10(3):R25. 19261174 10.1186/gb-2009-10-3-r25 PMC2690996

[evaa099-B34] Li B, Dewey CN. 2011 RSEM: accurate transcript quantification from RNA-Seq data with or without a reference genome. BMC Bioinformatics 12(1):323. 21816040 10.1186/1471-2105-12-323 PMC3163565

[evaa099-B35] Li H, Durbin R. 2009 Fast and accurate short read alignment with Burrows-Wheeler transform. Bioinformatics 25(14):1754–1760. 19451168 10.1093/bioinformatics/btp324 PMC2705234

[evaa099-B36] Li H, et al 2009 The sequence alignment/map format and SAMtools. Bioinformatics 25(16):2078–2079. 19505943 10.1093/bioinformatics/btp352 PMC2723002

[evaa099-B37] Lindholm A, Breden F. 2002 Sex chromosomes and sexual selection in poeciliid fishes. Am Nat. 160(S6):214–224. 18707478 10.1086/342898

[evaa099-B38] Lisachov AP, Zadesenets KS, Rubtsov NB, Borodin PM. 2015 Sex chromosome synapsis and recombination in male guppies. Zebrafish 12(2):174–180. 25608108 10.1089/zeb.2014.1000

[evaa099-B39] Lohse M, et al 2012 RobiNA: a user-friendly, integrated software solution for RNA-Seq-based transcriptomics. Nucleic Acids Res. 40(W1):622–627. 10.1093/nar/gks540 PMC3394330 22684630

[evaa099-B40] Löytynoja A, Goldman N. 2008 Phylogeny-aware gap replacement prevents errors in sequence alignment and evolutionary analysis. Science 320(5883):1632–1635. 18566285 10.1126/science.1158395

[evaa099-B41] Mank JE, Hosken DJ, Wedell N. 2011 Some inconvenient truths about sex chromosome dosage compensation and the potential role of sexual conflict. Evolution 65(8):2133–2144. 21790564 10.1111/j.1558-5646.2011.01316.x

[evaa099-B42] Martin H, et al 2019 Evolution of young sex chromosomes in two dioecious sister plant species with distinct sex determination systems. Genome Biol Evol. 11(2):350–361. 30649306 10.1093/gbe/evz001 PMC6364797

[evaa099-B43] Meredith RW, Pires MN, Reznick DN, Springer MS. 2011 Molecular phylogenetic relationships and the coevolution of placentotrophy and superfetation in *Poecilia* (Poeciliidae: Cyprinodontiformes). Mol Phylogenet Evol. 59(1):148–157. 21292015 10.1016/j.ympev.2011.01.014

[evaa099-B44] Morris J, Darolti I, Bloch NI, Wright AE, Mank JE. 2018 Shared and species-specific patterns of nascent Y chromosome evolution in two guppy species. Genes (Basel) 9(5):238. 10.3390/genes9050238 PMC5977178 29751570

[evaa099-B45] Muyle A, et al 2012 Rapid de novo evolution of X chromosome dosage compensation in *Silene latifolia*, a plant with young sex chromosomes. PLoS Biol. 10(4):e1001308. 22529744 10.1371/journal.pbio.1001308 PMC3328428

[evaa099-B46] Muyle A, et al 2016 SEX-DETector: a probabilistic approach to study sex chromosomes in non-model organisms. Genome Biol Evol. 8(8):2530–2543. 27492231 10.1093/gbe/evw172 PMC5010906

[evaa099-B47] Muyle A, et al 2018 Genomic imprinting mediates dosage compensation in a young plant XY system. Nat Plants. 4(9):677–680. 30104649 10.1038/s41477-018-0221-y

[evaa099-B48] Muyle A, Shearn R, Marais G. 2017 The evolution of sex chromosomes and dosage compensation in plants. Genome Biol Evol. 9(3):627–645. 28391324 10.1093/gbe/evw282 PMC5629387

[evaa099-B49] Natri HM, Shikano T, Merila J. 2013 Progressive recombination suppression and differentiation in recently evolved neo-sex chromosomes. Mol Biol Evol. 30(5):1131–1144. 23436913 10.1093/molbev/mst035 PMC3670740

[evaa099-B50] Nicolas M, et al 2004 A gradual process of recombination restriction in the evolutionary history of the sex chromosomes in dioecious plants. PLoS Biol. 3(1):e4. 15630476 10.1371/journal.pbio.0030004 PMC536007

[evaa099-B51] Papadopulos AS, Chester M, Ridout K, Filatov DA. 2015 Rapid Y degeneration and dosage compensation in plant sex chromosomes. Proc Natl Acad Sci U S A. 112(42):13021–13026. 26438872 10.1073/pnas.1508454112 PMC4620866

[evaa099-B52] R Core Team. 2015 R: a language and environment for statistical computing. Vienna, Austria:R Foundation for Statistical Computing. Available from: https://www.R-project.org/. Accessed May 25, 2020.

[evaa099-B53] Roesti M, Moser D, Berner D. 2013 Recombination in the threespine stickleback genome–patterns and consequences. Mol Ecol. 22(11):3014–3027. 23601112 10.1111/mec.12322

[evaa099-B54] Ross MT, et al 2005 The DNA sequence of the human X chromosome. Nature 434(7031):325–337. 15772651 10.1038/nature03440 PMC2665286

[evaa099-B55] Rosser ZH, Balaresque P, Jobling MA. 2009 Gene conversion between the X chromosome and the male-specific region of the Y chromosome at a translocation hotspot. Am J Hum Genet. 85(1):130–134. 19576564 10.1016/j.ajhg.2009.06.009 PMC2706966

[evaa099-B56] Rovatsos M, Farkačová K, Altmanová M, Johnson Pokorná M, Kratochvíl L. 2019 The rise and fall of differentiated sex chromosomes in geckos. Mol Ecol. 28(12):3042–3052. 31063656 10.1111/mec.15126

[evaa099-B57] Sawyer SA. 1999 GENECONV: a computer package for the statistical detection of gene conversion. Distributed by the author, Department of Mathematics, Washington University in St. Louis. Available from: https://www.math.wustl.edu/~sawyer/geneconv/, last accessed May 25, 2020.

[evaa099-B58] Slattery JP, Murphy WJ, O’Brien SJ. 2000 Patterns of diversity among SINE elements isolated from three Y-chromosome genes in carnivores. Mol Biol Evol. 17(5):825–829. 10779543 10.1093/oxfordjournals.molbev.a026361

[evaa099-B59] Stamatakis A. 2014 RAxML version 8: a tool for phylogenetic analysis and post-analysis of large phylogenies. Bioinformatics 30(9):1312–1313. 24451623 10.1093/bioinformatics/btu033 PMC3998144

[evaa099-B60] Stock M, et al 2011 Ever-young sex chromosomes in European tree frogs. PLoS Biol. 9:e1001062. 21629756 10.1371/journal.pbio.1001062 PMC3100596

[evaa099-B61] Stock M, et al 2013 Low rates of X–Y recombination, not turnovers, account for homomorphic sex chromosomes in several diploid species of Palearctic green toads (*Bufo viridis* subgroup). J Evol Biol. 26(3):674–682. 23316809 10.1111/jeb.12086

[evaa099-B62] Tripathi N, et al 2009 Genetic linkage map of the guppy, *Poecilia reticulata*, and quantitative trait loci analysis of male size and colour variation. Proc Biol Sci. 276(1665):2195–2208. 19324769 10.1098/rspb.2008.1930 PMC2677598

[evaa099-B63] Trombetta B, Cruciani F, Underhill PA, Sellitto D, Scozzari R. 2010 Footprints of X-to-Y gene conversion in recent human evolution. Mol Biol Evol. 27(3):714–725. 19812029 10.1093/molbev/msp231

[evaa099-B64] Van Doorn GS, Kirkpatrick M. 2010 Transitions between male and female heterogamety caused by sex-antagonistic selection. Genetics 186(2):629–645. 20628036 10.1534/genetics.110.118596 PMC2954476

[evaa099-B65] Veltsos P, et al 2019 Early sex-chromosome evolution in the diploid dioecious plant *Mercurialis annua*. Genetics 212(3):815–835. 31113811 10.1534/genetics.119.302045 PMC6614902

[evaa099-B66] Winge Ö. 1922 One-sided masculine and sex-linked inheritance in *Lebistes reticulatus*. J Gen. 12(2):145–162.

[evaa099-B67] Winge Ö. 1927 The location of eighteen genes in *Lebistes reticulatus*. J Gen. 18(1):1–43.

[evaa099-B68] Wright AE, et al 2017 Convergent recombination suppression suggests role of sexual selection in guppy sex chromosome formation. Nat Commun. 8(1):14251. 28139647 10.1038/ncomms14251 PMC5290318

[evaa099-B69] Wright AE, Darolti I, et al 2019 On the power to detect rare recombination events. Proc Natl Acad Sci U S A. 116(26):12607–12608. 31213531 10.1073/pnas.1905555116 PMC6601268

[evaa099-B70] Wright AE, Harrison PW, Montgomery SH, Pointer MA, Mank JE. 2014 Independent stratum formation on the avian sex chromosomes reveals inter-chromosomal gene conversion and predominance of purifying selection on the W chromosome. Evolution 68(11):3281–3295. 25066800 10.1111/evo.12493 PMC4278454

[evaa099-B71] Wright AE, Moghadam HK, Mank JE. 2012 Trade-off between selection for dosage compensation and masculinization on the avian Z chromosome. Genetics 192(4):1433–1445. 22997237 10.1534/genetics.112.145102 PMC3512148

[evaa099-B72] Wright AE, Rogers TF, Fumagalli M, Cooney CR, Mank JE. 2019 Phenotypic sexual dimorphism is associated with genomic signatures of resolved sexual conflict. Mol Ecol. 28(11):2860–2871. 31038811 10.1111/mec.15115 PMC6618015

[evaa099-B73] Yamamoto T. 1975 The medaka, *Oryzias latipes*, and the guppy, *Lebistes reticularis* In: King RC, editor. Handbook of Genetics. Boston: Springer p. 133–149.

[evaa099-B74] Yang Z. 2007 PAML 4: phylogenetic analysis by maximum likelihood. Mol Biol Evol. 24(8):1586–1591. 17483113 10.1093/molbev/msm088

[evaa099-B75] Yang Z, Nielsen R. 2000 Estimating synonymous and nonsynonymous substitution rates under realistic evolutionary models. Mol Biol Evol. 17(1):32–43. 10666704 10.1093/oxfordjournals.molbev.a026236

