# Supplementary material for: Guppy Y Chromosome Integrity Maintained by Incomplete Recombination Suppression
Source: Genome Biol Evol. 2020 May 19;12(6):965–77. doi: 10.1093/gbe/evaa099 (PMC7337182; doi:10.1093/gbe/evaa099)

SUPPORTING INFORMATION

Manuscript title: Guppy Y chromosome integrity maintained by incomplete recombination suppression

Authors: Iulia Darolti[1], Alison E. Wright[2], Judith E. Mank[1,3]

Affiliations: 1 Department of Zoology, University of British Columbia, Canada; 2 Department of Animal and Plant Sciences, University of Sheffield, United Kingdom; 3 Department of Genetics, Evolution and Environment, University College London, United Kingdom

8 SI RESULTS

Supplementary Table 1. Sequencing information for each sample

Species (Treatment)	Family Number	Sample	Raw paired reads (Million)	Paired reads after trimming (Million)	% kept
P. reticulata (RNA-seq PE)	Family 1	Father	42.3	29.1	68.8
		Mother	34.7	21.8	62.8
		Off. Fem. 1	29.5	20.1	68.1
		Off. Fem. 2	135.4	88.2	65.1
		Off. Fem. 3	27.9	23.3	83.5
		Off. Fem. 4	33.4	20.8	62.3
		Off. Fem. 5	25.2	17.6	69.8
		Off. Mal. 1	18.6	12.3	66.1
		Off. Mal. 2	20.3	14.0	69.0
		Off. Mal. 3	42.8	27.2	63.6
		Off. Mal. 4	47.6	35.7	75.0
		Off. Mal. 5	59.2	43.3	73.1
	Family 2	Father	52.2	33.8	64.8
		Mother	86.6	58.4	67.4
		Off. Fem. 1	24.5	16.4	66.9
		Off. Fem. 2	35.8	23.4	65.4
		Off. Fem. 3	53.5	36.7	68.6
		Off. Fem. 4	27.7	18.9	68.2
		Off. Fem. 5	57.4	35.9	62.5
		Off. Mal. 1	72.7	46.7	64.2
		Off. Mal. 2	19.5	12.7	65.1
		Off. Mal. 3	76.3	51.7	67.8
		Off. Mal. 4	33.1	24.4	73.7
		Off. Mal. 5	25.4	16.6	65.4
	Family 3	Father	28.1	23.5	83.6
		Mother	33.2	19.8	59.6
		Off. Fem. 1	35.2	21.6	61.4
		Off. Fem. 2	23.5	15.5	66.0
		Off. Fem. 3	50.2	31.1	62.0
		Off. Fem. 4	97.9	63.4	64.8
		Off. Fem. 5	30.5	20.6	67.5
		Off. Mal. 1	39.9	26.8	67.2
		Off. Mal. 2	45.1	26.9	59.6
		Off. Mal. 3	26.0	22.3	85.8
		Off. Mal. 4	30.6	25.6	83.7
		Off. Mal. 5	35.7	22.7	63.6
P. wingei (RNA-seq PE)	Family 1	Father	25.4	15.3	60.2
		Mother	92.0	58.8	63.9
		Off. Fem. 1	29.9	22.7	75.9
		Off. Fem. 2	30.6	21.2	69.3

	Off. Fem. 3	32.1	24.3	75.7
	Off. Fem. 4	26.1	17.7	67.8
	Off. Fem. 5	31.6	23.8	75.3
	Off. Mal. 1	34.0	24.9	73.2
	Off. Mal. 2	22.0	15.4	70.0
	Off. Mal. 3	58.7	41.9	71.4
	Off. Mal. 4	48.1	34.1	70.9
	Off. Mal. 5	43.9	27.3	62.2
Family 2	Father	54.1	35.7	66.0
	Mother	52.0	35.0	67.3
	Off. Fem. 1	25.6	16.3	63.7
	Off. Fem. 2	31.4	27.9	88.9
	Off. Fem. 3	54.8	29.1	53.1
	Off. Fem. 4	26.0	16.7	64.2
	Off. Fem. 5	29.1	19.3	66.3
	Off. Mal. 1	48.2	30.1	62.5
	Off. Mal. 2	46.1	30.0	65.1
	Off. Mal. 3	71.2	49.0	68.8
	Off. Mal. 4	36.1	24.9	69.0
	Off. Mal. 5	34.2	23.6	69.0

Supplementary Table 2. ***De novo*** **transcriptome assembly statistics**

		Before filtering	After best isoform selection	After ncRNA filter	After ORF filter	After CAP3
P. reticulata (n = 36)	No. transcripts	490,973	249,752	249,528	21,141	19,935
	N50	2,511	1,050	1,048	1,713	1,821
	Median length	548	357	357	1,041	1,092
P. wingei (n = 24)	No. transcripts	408,978	209,871	209,682	20,340	19,361
	N50	2,730	1,150	1,148	1,749	1,845
	Median length	554	355	355	1,074	1,119

Supplementary Table 3. Genomic distribution of inferred sex-linked genes

	Linkage Groups	*P. reticulata*	*P. wingei*
	LG1	0	1
	LG2	0	1
	LG3	0	0
	LG4	0	0
	LG5	0	0
	LG6	1	1
	LG7	1	2
	LG8	0	1
	LG9	0	1
	LG10	1	2
Autosomes	LG11	0	1
	LG13	0	0
	LG14	0	0
	LG15	3	1
	LG16	1	0
	LG17	3	0
	LG18	1	0
	LG19	0	0
	LG20	0	0
	LG21	2	0
	LG22	0	0
	LG23	0	1
Sex chromosomes	LG12	92	249
	NW_007615023.1	0	2
	NW_007615029.1	3	2
	NW_007615033.1	0	1
	NW_007615064.1	1	2
Unplaced scaffolds*	NW_007615133.1	1	0
	NW_007615235.1	0	1
	NW_007615409.1	1	0
	NW_007615432.1	0	1
	NW_007617302.1	0	1
	NW_007617506.1	0	1

*List only includes scaffolds containing genes with a sex-linked segregation pattern in at least one of the species

Supplementary Table 4. Relevant molecular functions and biological processes associated with the 42 shared sex-linked genes between *P. reticulata* and *P. wingei*. Genes highlighted in green are the four sex-linked genes showing clustering by chromosome type.

Gene name	LG12 position (Mb)	Gene description	Relevant functions and processes*
LOC103473567	2.6	claudin-23-like	NA
noc4l	3.8	nucleolar complex associated 4 homolog	ribosome biogenesis
nup155	7.1	nucleoporin 155	structural constituent of nuclear pore, nucleocytoplasmic transport
fbxw5	7.9	F-box and WD repeat domain containing 5	protein binding, protein ubiquitination
nup214	9.3	nucleoporin 214	protein binding
prrc2b	9.4	proline rich coiled-coil 2B	NA
dym	10.0	dymeclin	NA
prlr	10.1	prolactin receptor	cytokine receptor activity, prolactin receptor activity, protein binding, metal ion binding, prolactin signalling pathway
dnajc21	10.2	DnaJ heat shock protein family (Hsp40) member C21	nucleic acid binding, zinc ion binding, metal ion binding
arhgap24	10.6	Rho GTPase activating protein 24	GTPase activator activity, signal transduction, positive regulation of GTPase activity
sdad1	10.8	SDA1 domain containing 1	ribosomal large subunit export from nucleus, protein transport, actin cytoskeleton organization, ribosome biogenesis, ribosomal large subunit biogenesis
skiv2l2	11.1	Mtr4 exosome RNA helicase	nucleic acid binding, RNA binding, RNA helicase activity, ATP binding, RNA catabolic process, fin regeneration positive regulation of cell division
sec16a	11.6	SEC16 homolog A, endoplasmic reticulum export factor	protein transport, vesicle-mediated transport
LOC103473427	12.0	rap guanine nucleotide exchange factor 1-like	guanyl-nucleotide exchange factor activity, small GTPase mediated signal transduction
uck1	12.0	uridine-cytidine kinase 1	nucleotide binding, uridine kinase activity, ATP binding, kinase activity, transferase activity, nucleotide biosynthetic process, phosphorylation
ppp1r26	13.5	protein phosphatase 1 regulatory subunit 26	protein phosphatase inhibitor activity
LOC103473338	15.2	tumor necrosis factor receptor superfamily member 10B-like	protein binding, signal transduction, positive regulation of apoptotic process
trappc13	15.2	trafficking protein particle complex 13	NA
LOC103473319	15.7	zinc finger protein 703-like	NA
dmgdh	15.9	dimethylglycine dehydrogenase	protein binding, oxidoreductase activity, oxidation-reduction process
ap3b1	16.1	adaptor related protein complex 3 subunit beta 1	intracellular protein transport, vesicle-mediated transport

LOC103473499	16.3	proteinase-activated receptor 1-like	G protein-coupled receptor activity, thrombin-activated receptor activity, signal transduction, blood coagulation
LOC103473209	17.9	N-acetylglucosamine-6-sulfatase-like	catalytic activity, hydrolase activity, glycosaminoglycan metabolic process
LOC103473177	18.7	multiple C2 and transmembrane domain-containing protein 1-like	NA
LOC103473119	20.1	hydroxymethylglutaryl-CoA synthase, cytoplasmic-like	catalytic activity, hydroxymethylglutaryl-CoA synthase activity, embryonic viscerocranium morphogenesis, transferase activity, lipid metabolic process steroid biosynthetic and metabolic process, neural crest cell differentiation
kiaa0020	20.9	pumilio RNA binding family member 3	NA
LOC103473940	21.1	hippocampus abundant transcript 1 protein-like	NA
dnajc25	21.1	DnaJ (Hsp40 - Heat shock protein) homolog	NA
LOC103473919	21.3	cyclin-G2-like	regulation of cell cycle
ccni	21.4	cyclin I	regulation of cell cycle
alad	21.7	aminolevulinate dehydratase	catalytic activity, porphobilinogen synthase activity, metal ion binding, tetrapyrrole biosynthetic process
npr2	21.7	atrial natriuretic peptide receptor 2	nucleotide binding, guanylate cyclase activity, protein kinase activity, ATP binding, lyase activity, cGMP biosynthetic process, protein phosphorylation, intracellular signal transduction, cardiac muscle cell proliferation
LOC103473957	22.2	syntaxin-binding protein 1	vesicle docking involved in exocytosis, vesicle-mediated transport
LOC103473998	22.4	ectonucleoside triphosphate diphosphohydrolase 2-like	NA
tsc1	22.6	TSC complex subunit 1	NA
LOC103473971	22.8	gelsolin-like	calcium ion binding, actin filament binding, actin nucleation, actin filament severing
LOC103473970	22.8	erythrocyte band 7 integral membrane protein-like	NA
LOC103473986	24.4	5'-AMP-activated protein kinase subunit	NA
LOC103474035	24.8	lisH domain-containing protein	NA
LOC103474042	24.9	prolactin receptor-like	NA
pigo	25.3	phosphatidylinositol glycan anchor biosynthesis class O	catalytic activity, transferase activity, mannose-ethanolamine phosphotransferase activity, GPI anchor biosynthetic process
slc30a5	25.8	solute carrier family 30 member 5	NA

*Compiled from Ensembl Biomart

Supplementary Table 5. Contingency tables comparing the proportion of *P. reticulata* sex-linked and autosomal genes on the total non-recombining region, Stratum II and Stratum I relative to that on the pseudoautosomal region, using Fisher's Exact Test.

	Sex-linked genes	Autosomal genes	*p* value	Odds ratio
**PAR (0-15Mb, 26-27Mb)**	32 (36.4%)	56 (63.6%)		
**Total non-recombining region (15-26Mb)**	60 (63.2%)	35 (36.8%)	**0.0004**	2.98
**Stratum II (15-21Mb)**	18 (62.1%)	11 (37.9%)	**0.0011**	3.04
**Stratum I (21-26Mb)**	42 (63.6%)	24 (36.4%)	**0.0183**	2.84

Significant *p* values are shown in bold.

Supplementary Table 6. Mean divergence estimates for *P. reticulata* and *P. wingei* X- and Y-linked gametologs. Nonsynonymous (*d*N) and synonymous (*d*S) substitutions for X- and Y-linked sequences were estimated using branch model 2 from the CODEML package in PAML. Bootstrapping with 1,000 replicates was used to determine the confidence intervals shown in brackets. Significance *p* values are based on 1,000 permutation test replicates comparing substitution estimates between X and Y sequences of each species.

		**X**	**Y**	**sig.***	**X-Y**
	***d*N**	0.0002 (0.0000–0.0008)	0.0004 (0.0002–0.0006)	0.914	0.0005 (0.0004)
P. reticulata	***dS***	0.0045 (0.0027-0.0086)	0.0044 (0.0031–0.0063)	0.106	0.0059 (0.0047)
	***d*N/*d*S**	0.0410 (0.0000–0.1395)	0.0869 (0.0444–0.1273)	0.102	0.0869 (0.0810)
	***d*N**	0.0003 (0.0001–0.0006)	0.0003 (0.0001–0.0006)	0.464	0.0024 (0.0008)
P. wingei	***dS***	0.0050 (0.0033–0.0074)	0.0045 (0.0026–0.0077)	0.978	0.0143 (0.0063)
	***d*N/*d*S**	0.0651 (0.0126–0.1489)	0.0722 (0.0357–0.1324)	0.528	0.1665 (0.1268)

Supplementary Figure 1. Synteny between the *P. reticulata*, *P. wingei* and *P. picta* sex chromosomes. Shown is the orientation of scaffolds mapping to *P. reticulata* chromosome 12. Regions showing the same orientation are highlighted in blue, inverted regions in green. The inversion is specific to the inbred strain on which the *P. reticulata* reference genome assembly was built. We have accounted for this inversion when assigning genes with position on *P. reticulata* chromosomes.

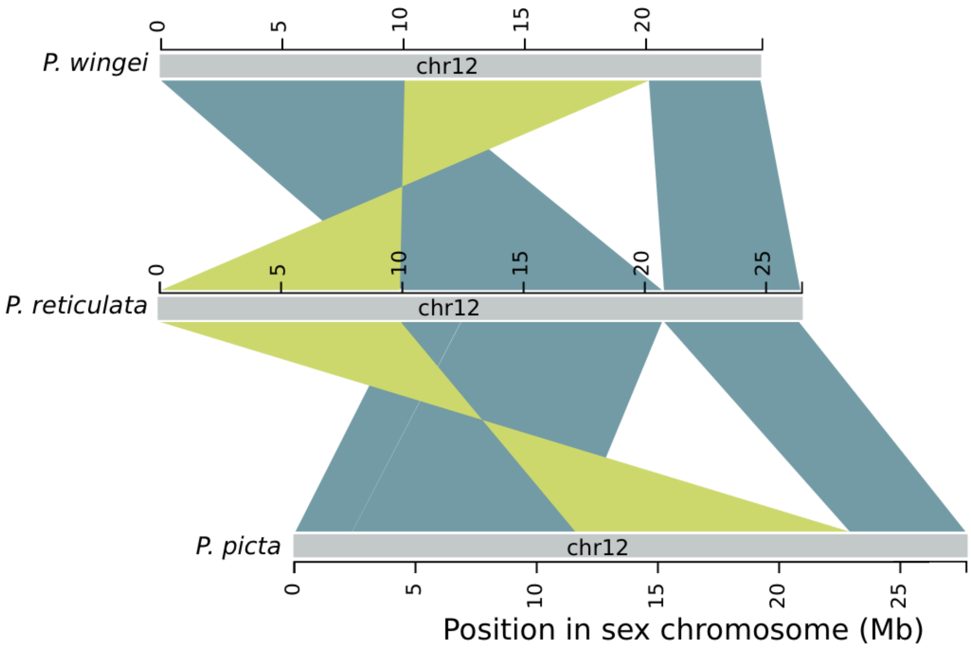

Supplementary Figure 2. Density of sex-linked genes across the sex chromosome in each of the *P. reticulata* (A) and *P. wingei* (B) families. The shaded purple regions indicate the identified non-recombining regions. Stratum I is shown in dark purple, where X-Y divergence is the greatest, and Stratum II is shown in light purple.

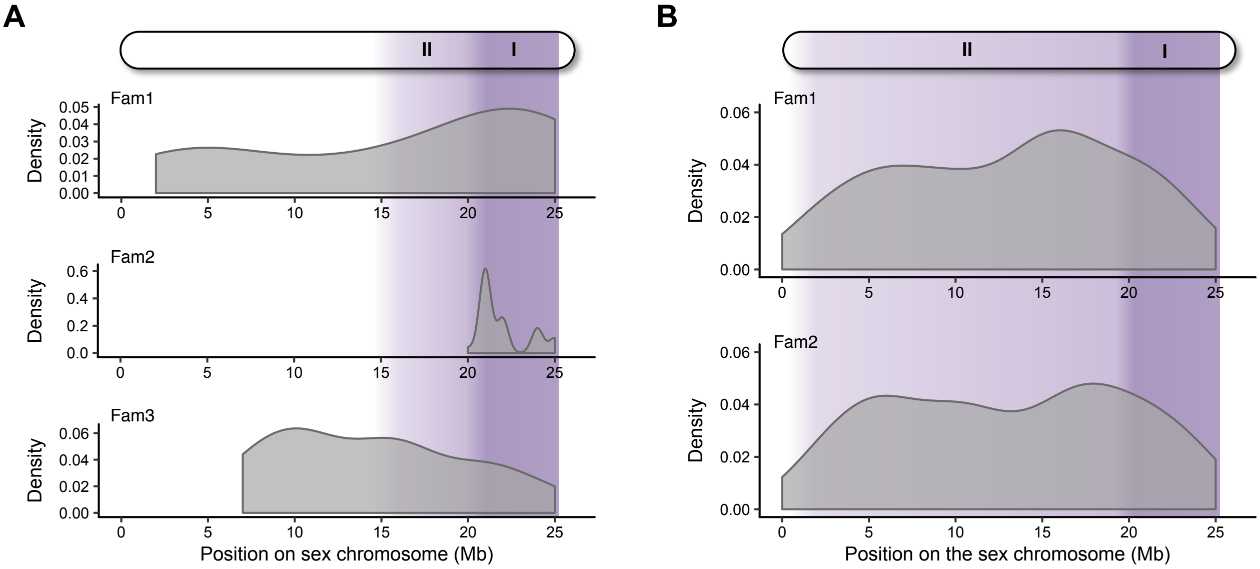

Supplementary Figure 3. The number of sex-linked (purple) and autosomal (grey) genes across the *P. reticulata* and *P. wingei* sex chromosomes.

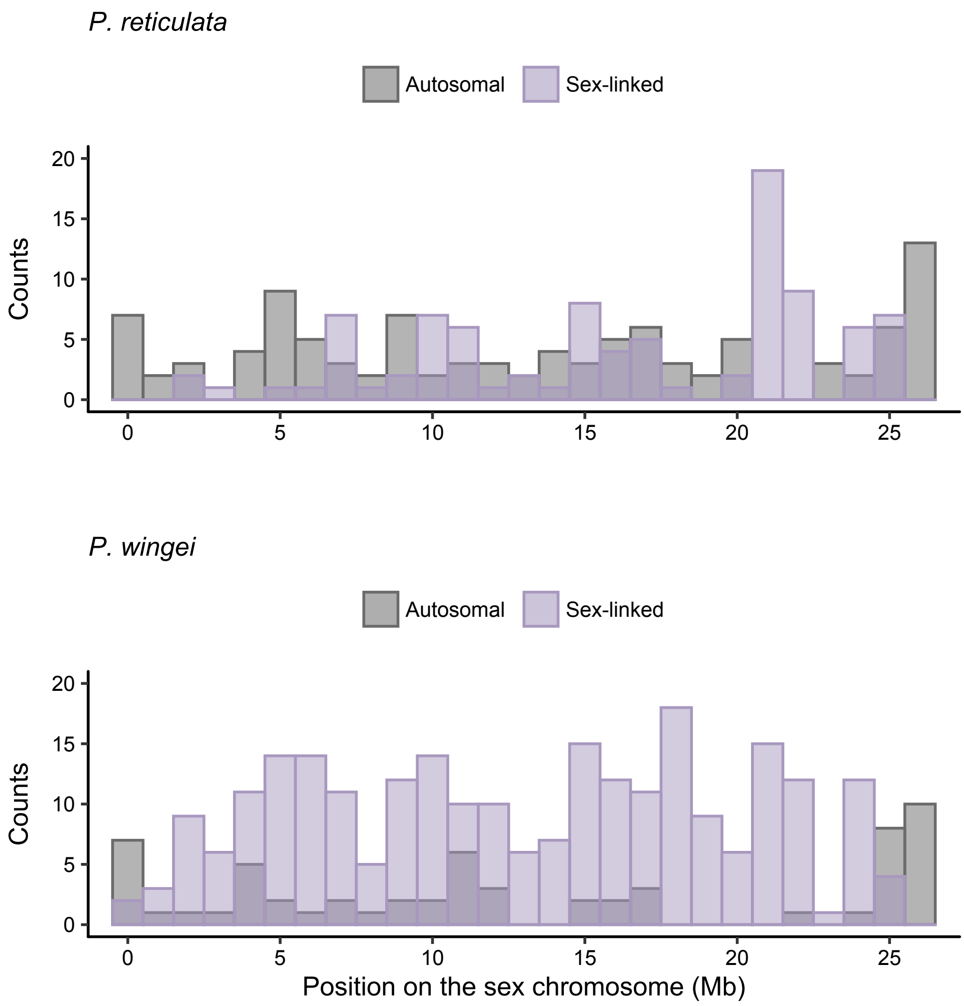

Supplementary Fig. 4. Phylogenetic gene trees for *P. reticulata* and *P. wingei* X- and Y-linked sequences based on the MEGA analysis. Phylogenetic trees for the four sex-linked genes in which the X (red) and Y (blue) sequences cluster by gametolog instead of by species. (A) Consensus tree based on alignments of all four sex-linked genes. Numbers at each node represent bootstrap values based on 100 permutations. Branches with the interrupted lines have been shortened to improve clarity. (B) *alad* (C) *dnajc25* (D) *LOC103473940* (E) *LOC103474035*.

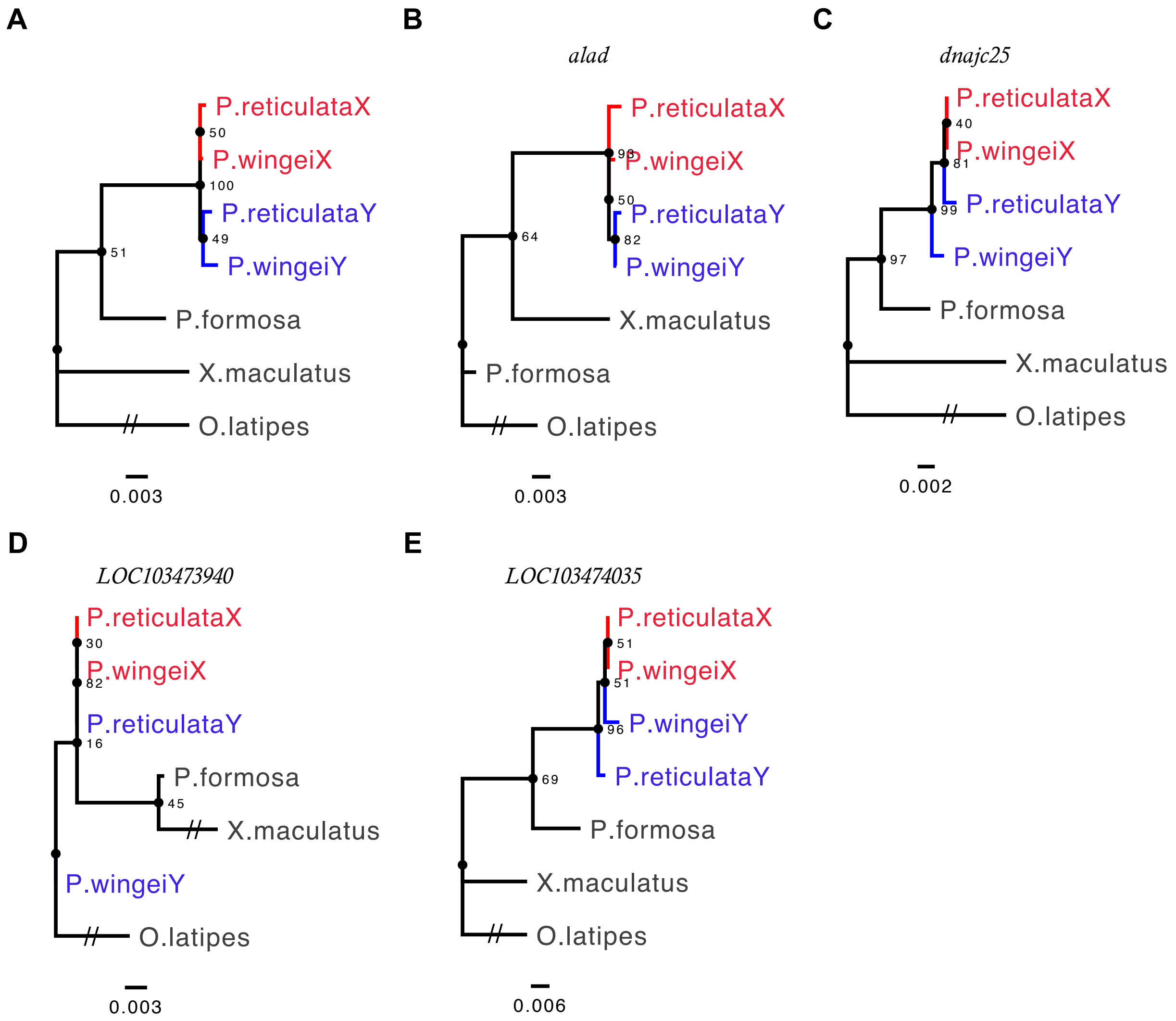

Supplementary Figure 5. Majority consensus phylogenetic tree based on alignments of all sex-linked genes. X- and Y-linked sequences are highlighted in red and blue, respectively. Numbers at each node represent bootstrap values based on 100 permutations. The *O. latipes* branch has been shortened to improve clarity.

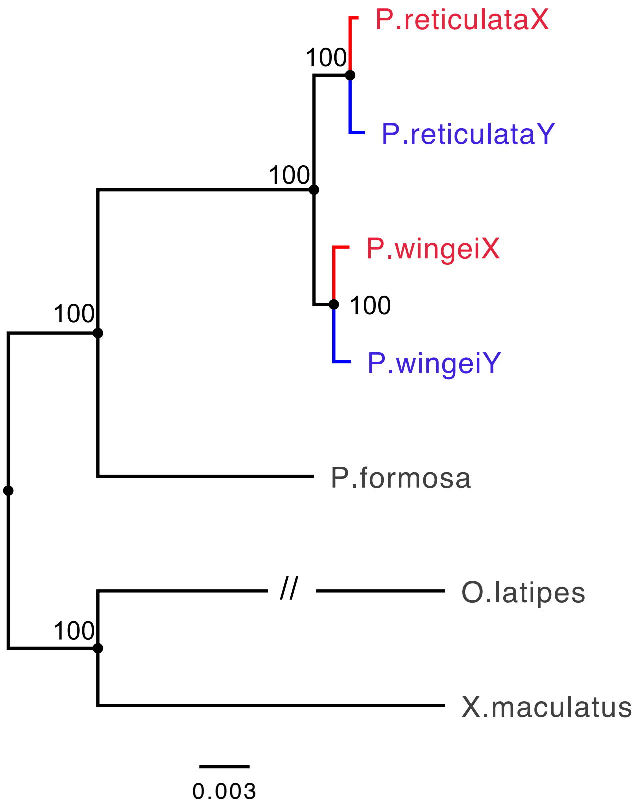

Supplementary Figure 6. Phylogenetic gene trees of *P. reticulata* and *P. wingei* X- and Y-linked sequences for the 38 identified sex-linked genes showing clustering by species. Numbers at each node represent bootstrap values based on 100 permutations. Branches with the interrupted lines have been shortened to improve clarity.

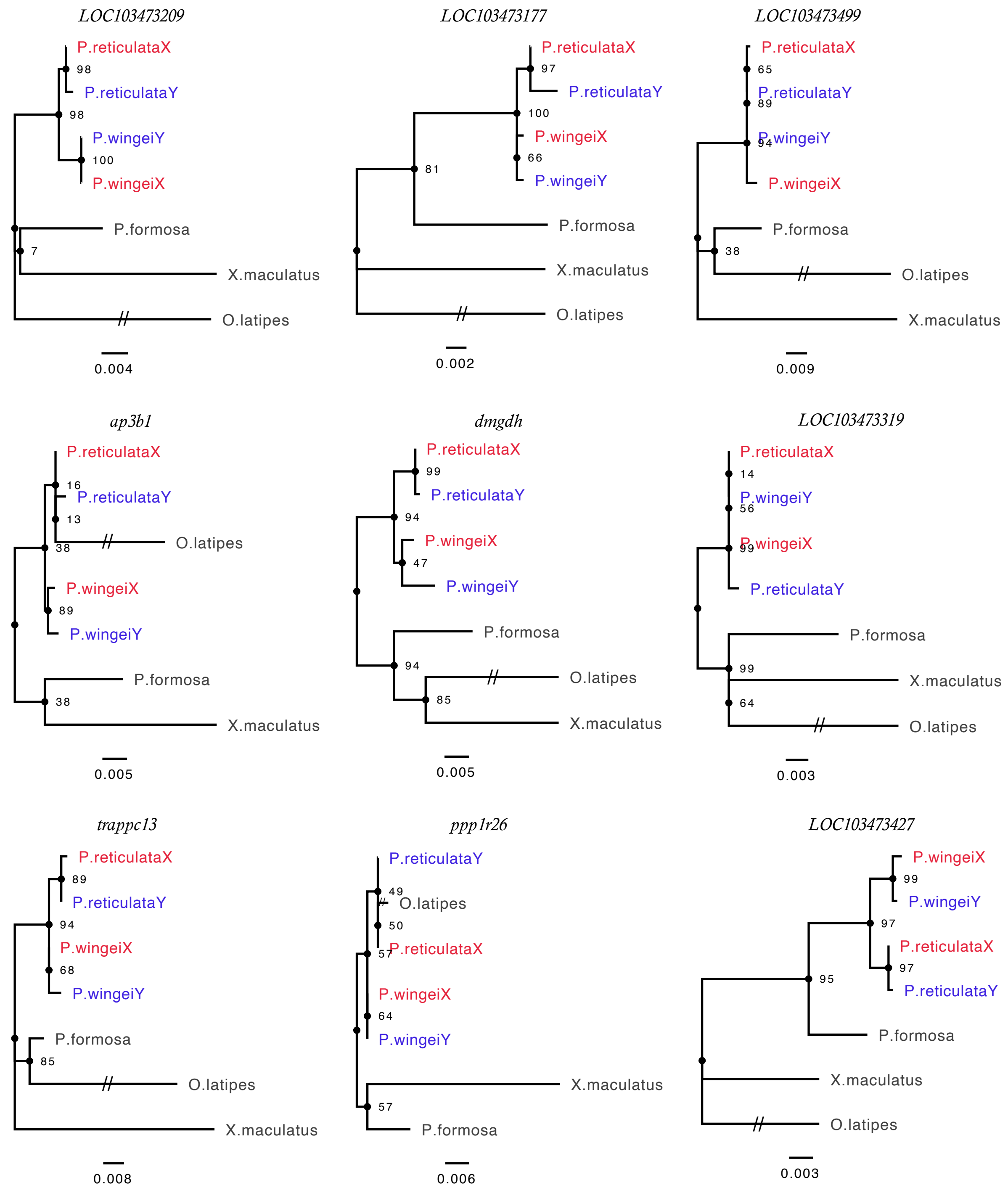

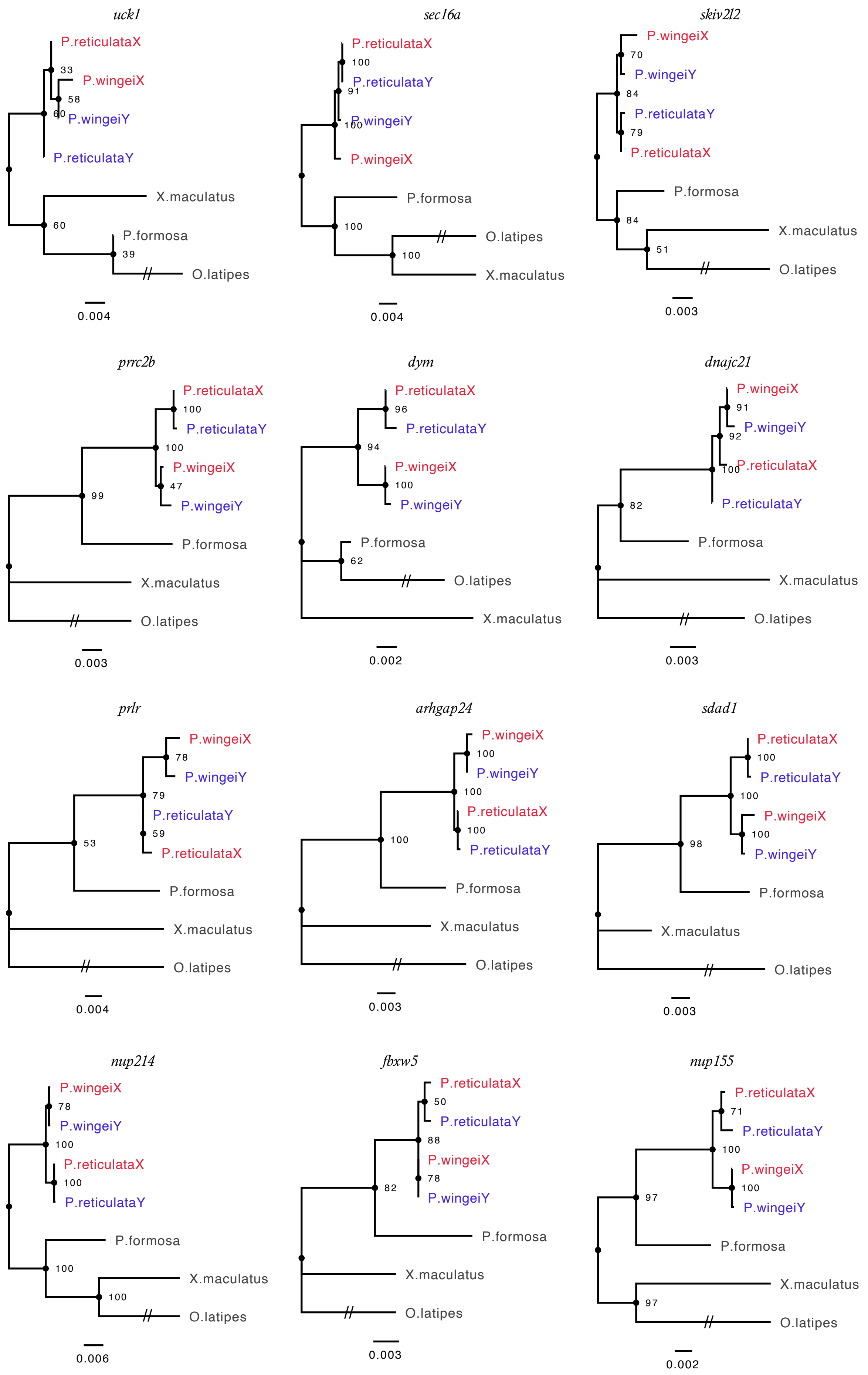
uck1
P.reticulataX
33
P.wingeiX
58
60
P.wingeiY
P.reticulataY
X.maculatus
60
P.formosa
39
O.latipes
0.004
sec16a
P.reticulataX
100
P.reticulataY
91
100
P.wingeiY
P.wingeiX
P.formosa
100
O.latipes
100
X.maculatus
0.004
skiv2l2
P.wingeiX
70
P.wingeiY
84
P.reticulataY
79
P.reticulataX
P.formosa
84
X.maculatus
51
O.latipes
0.003
prrc2b
P.reticulataX
100
P.reticulataY
100
P.wingeiX
47
P.wingeiY
99
P.formosa
X.maculatus
O.latipes
0.003
dym
P.reticulataX
96
P.reticulataY
94
P.wingeiX
100
P.wingeiY
P.formosa
62
O.latipes
X.maculatus
0.002
dnajc21
P.wingeiX
91
P.wingeiY
92
100
P.reticulataX
P.reticulataY
82
P.formosa
X.maculatus
O.latipes
0.003
prlr
P.wingeiX
78
P.wingeiY
79
P.reticulataY
59
P.reticulataX
53
P.formosa
X.maculatus
O.latipes
0.004
arhgap24
P.wingeiX
100
P.wingeiY
100
P.reticulataX
100
P.reticulataY
100
P.formosa
X.maculatus
O.latipes
0.003
sdad1
P.reticulataX
100
P.reticulataY
100
P.wingeiX
100
P.wingeiY
98
P.formosa
X.maculatus
O.latipes
0.003
nup214
P.wingeiX
78
P.wingeiY
100
P.reticulataX
100
P.reticulataY
P.formosa
100
X.maculatus
100
O.latipes
0.006
fbxw5
P.reticulataX
50
P.reticulataY
88
P.wingeiX
78
P.wingeiY
82
P.formosa
X.maculatus
O.latipes
0.003
nup155
P.reticulataX
71
P.reticulataY
100
P.wingeiX
100
P.wingeiY
97
P.formosa
X.maculatus
97
O.latipes
0.002

60

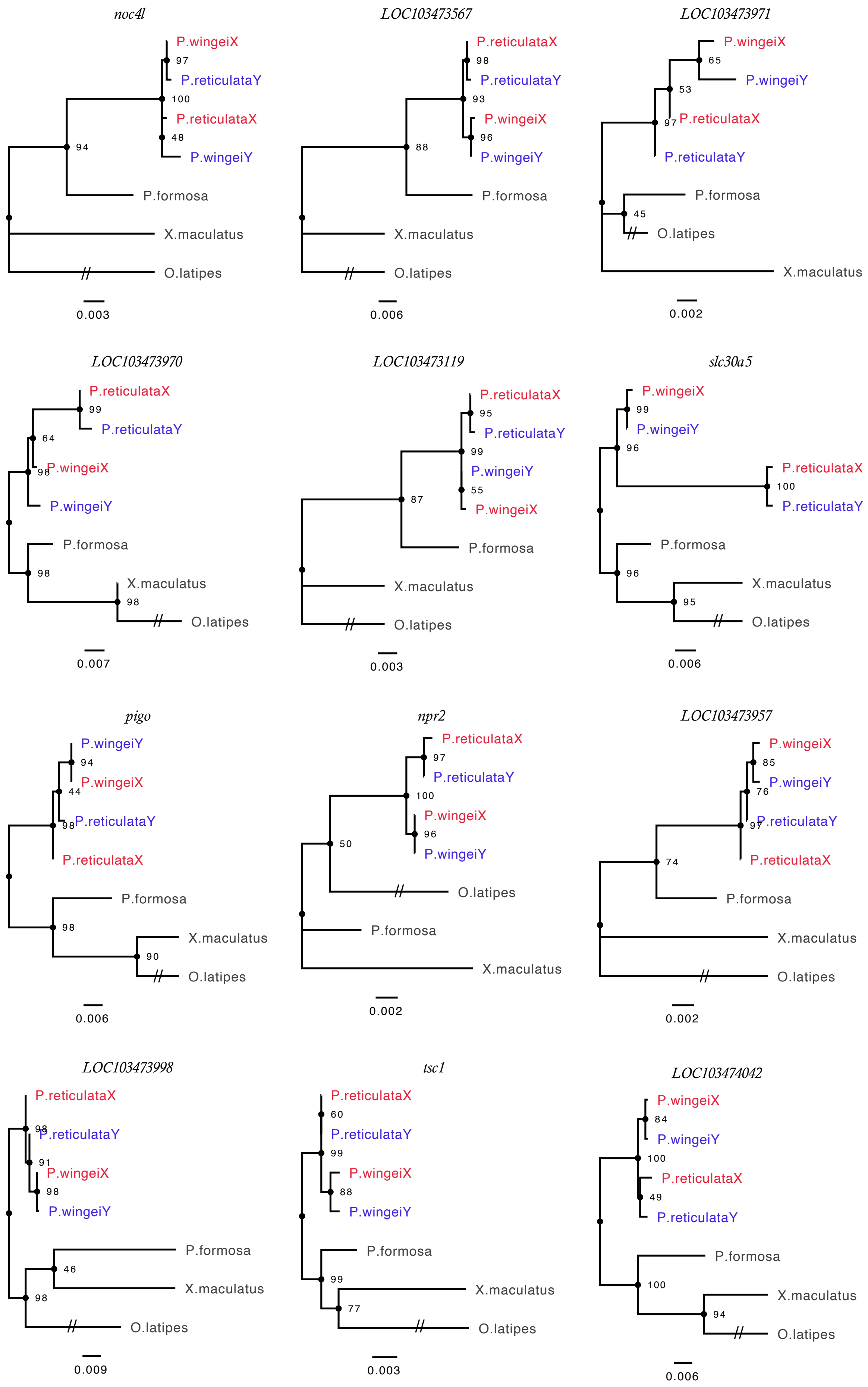
noc4l
P.wingeiX
97
P.reticulataY
100
P.reticulataX
48
P.wingeiY
94
P.formosa
X.maculatus
O.latipes
0.003
LOC103473567
P.reticulataX
98
P.reticulataY
93
P.wingeiX
96
P.wingeiY
88
P.formosa
X.maculatus
O.latipes
0.006
LOC103473971
P.wingeiX
65
P.wingeiY
53
97
P.reticulataX
P.reticulataY
P.formosa
45
O.latipes
X.maculatus
0.002
LOC103473970
P.reticulataX
99
P.reticulataY
64
98
P.wingeiX
P.wingeiY
P.formosa
98
X.maculatus
98
O.latipes
0.007
LOC103473119
P.reticulataX
95
P.reticulataY
99
P.wingeiY
55
P.wingeiX
87
P.formosa
X.maculatus
O.latipes
0.003
slc30a5
P.wingeiX
99
P.wingeiY
96
P.reticulataX
100
P.reticulataY
P.formosa
96
X.maculatus
95
O.latipes
0.006
pigo
P.wingeiY
94
P.wingeiX
44
98
P.reticulataY
P.reticulataX
P.formosa
98
X.maculatus
90
O.latipes
0.006
npr2
P.reticulataX
97
P.reticulataY
100
P.wingeiX
96
P.wingeiY
50
O.latipes
P.formosa
X.maculatus
0.002
LOC103473957
P.wingeiX
85
P.wingeiY
76
97
P.reticulataY
P.reticulataX
74
P.formosa
X.maculatus
O.latipes
0.002
LOC103473998
P.reticulataX
98
P.reticulataY
91
P.wingeiX
98
P.wingeiY
P.formosa
46
X.maculatus
98
O.latipes
0.009
tsc1
P.reticulataX
60
P.reticulataY
99
P.wingeiX
88
P.wingeiY
P.formosa
99
X.maculatus
77
O.latipes
0.003
LOC103474042
P.wingeiX
84
P.wingeiY
100
P.reticulataX
49
P.reticulataY
P.formosa
100
X.maculatus
94
O.latipes
0.006

61

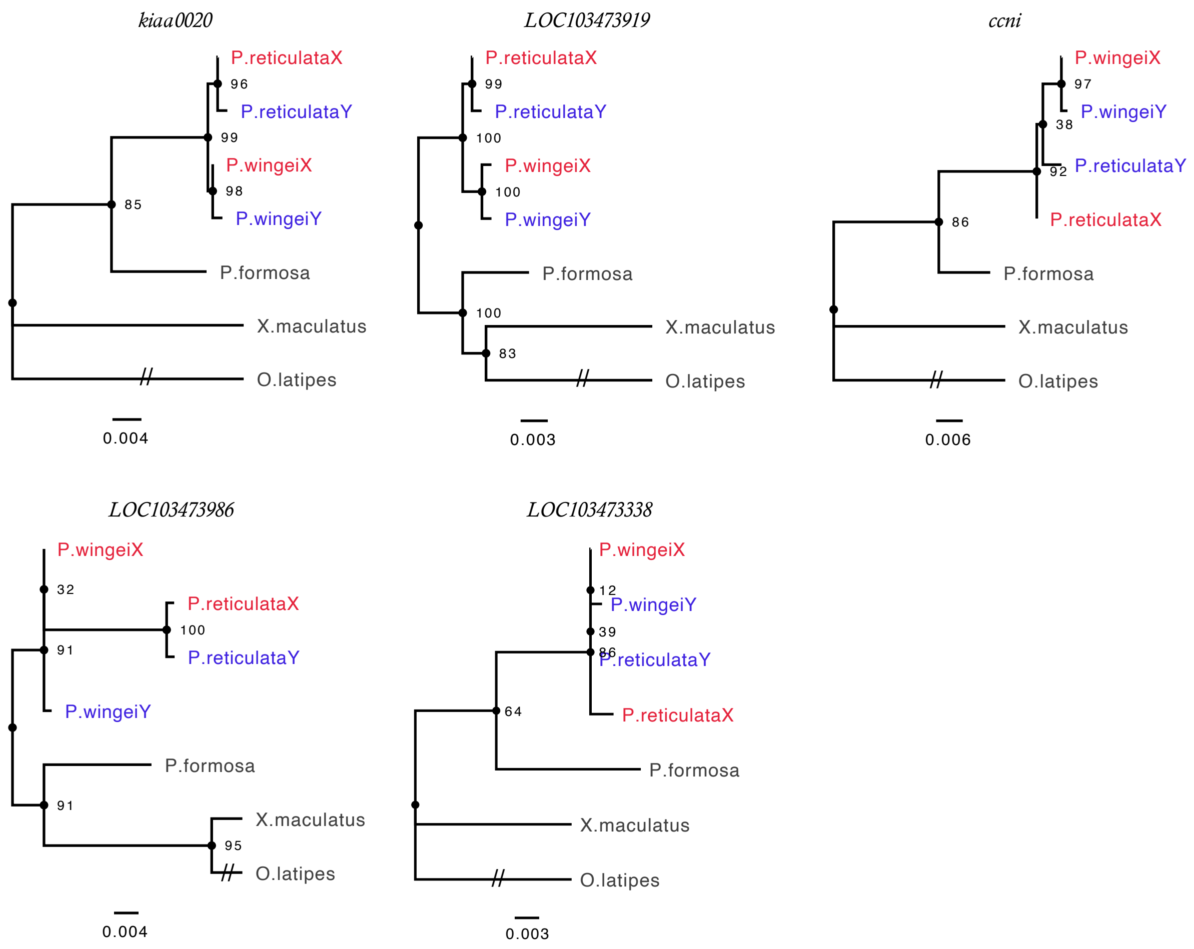

kiaa0020
P.reticulataX
96
P.reticulataY
99
P.wingeiX
98
P.wingeiY
85
P.formosa
X.maculatus
O.latipes
0.004
LOC103473919
P.reticulataX
99
P.reticulataY
100
P.wingeiX
100
P.wingeiY
P.formosa
100
X.maculatus
83
O.latipes
0.003
ccni
P.wingeiX
97
P.wingeiY
38
P.reticulataY
92
P.reticulataX
86
P.formosa
X.maculatus
O.latipes
0.006
LOC103473986
P.wingeiX
32
P.reticulataX
100
91
P.reticulataY
P.wingeiY
P.formosa
91
X.maculatus
95
O.latipes
0.004
LOC103473338
P.wingeiX
12
P.wingeiY
39
86
P.reticulataY
P.reticulataX
64
P.formosa
X.maculatus
O.latipes
0.003

Supplementary Figure 7. Distribution of pairwise synonymous divergence estimates between X and Y gametologs.

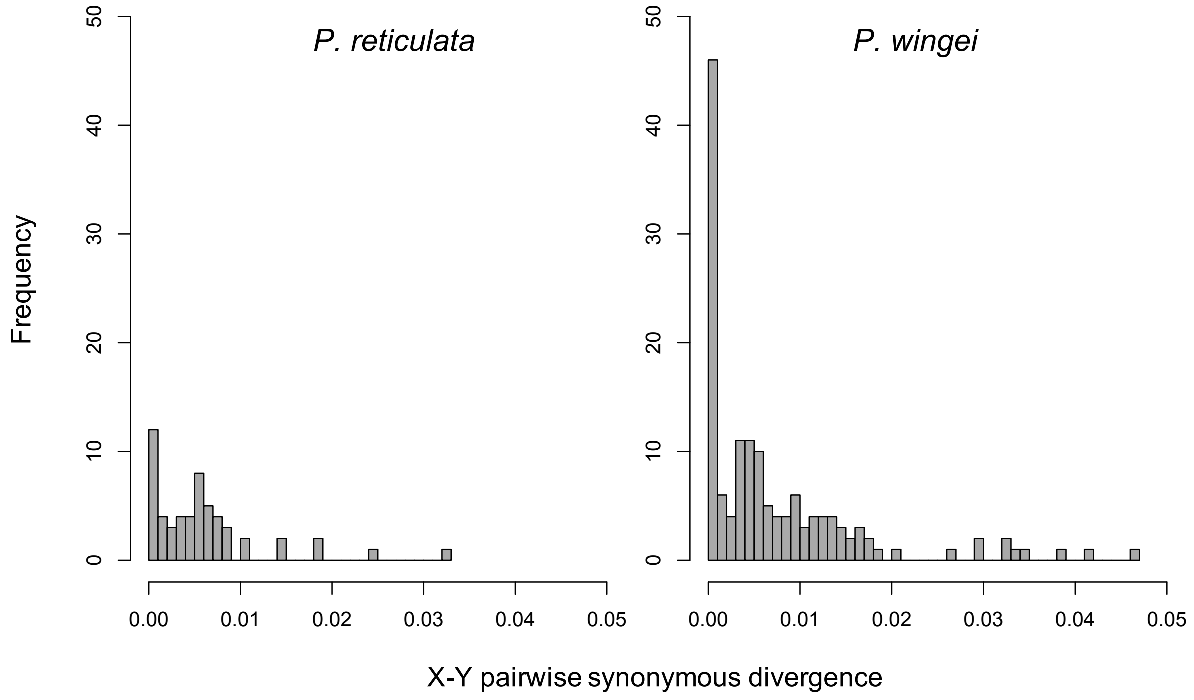

Supplement: evaa099_Supplementary_Data [file evaa099_supplementary_data.pdf]
